# The histone demethylase Kdm6b regulates the maturation and cytotoxicity of TCRαβ^+^CD8αα^+^ intestinal intraepithelial lymphocytes

**DOI:** 10.1038/s41418-021-00921-w

**Published:** 2022-01-09

**Authors:** Haohao Zhang, Yiming Hu, Dandan Liu, Zhi Liu, Ningxia Xie, Sanhong Liu, Jie Zhang, Yuhang Jiang, Cuifeng Li, Qi Wang, Xi Chen, Deji Ye, Donglin Sun, Yujia Zhai, Xinhui Yan, Yongzhong Liu, Charlie Degui Chen, Xingxu Huang, Y. Eugene Chin, Yufang Shi, Baojin Wu, Xiaoren Zhang

**Affiliations:** 1 Affiliated Cancer Hospital and Institute of Guangzhou Medical University; Key Laboratory for Cell Homeostasis and Cancer Research of Guangdong Higher Education Institutes; State Key Laboratory of Respiratory Disease, 510000 Guangzhou, China; 2 CAS Key Laboratory of Tissue Microenvironment and Tumor, Shanghai Institute of Nutrition and Health, Shanghai Institutes for Biological Sciences, University of Chinese Academy of Sciences, Chinese Academy of Sciences, 200031 Shanghai, China; 3 Institute of Interdisciplinary Integrative Medicine Research, Shanghai University of Traditional Chinese Medicine, 201203 Shanghai, China; 4 State Key Laboratory of Oncogenes and Related Genes, Shanghai Cancer Institute, Renji Hospital, Shanghai Jiao Tong University School of Medicine, 200032 Shanghai, China; 5 State Key Laboratory of Molecular Biology, Shanghai Key Laboratory of Molecular Andrology, CAS Center for Excellence in Molecular Cell Science, Shanghai Institute of Biochemistry and Cell Biology, University of Chinese Academy of Sciences, Chinese Academy of Sciences, 200031 Shanghai, China; 6 Institutes of Biology and Medical Sciences, Soochow University Medical College, 215000 Suzhou, China; 7 Shanghai Institute of Biochemistry and Cell Biology, Center for Excellence in Molecular Cell Science, Chinese Academy of Sciences, 200031 Shanghai, China

**Keywords:** Epigenetics, Gene regulation, Immunological disorders, T cells

## Abstract

Intestinal intraepithelial lymphocytes (IELs) are distributed along the length of the intestine and are considered the frontline of immune surveillance. The precise molecular mechanisms, especially epigenetic regulation, of their development and function are poorly understood. The trimethylation of histone 3 at lysine 27 (H3K27Me3) is a kind of histone modifications and associated with gene repression. Kdm6b is an epigenetic enzyme responsible for the demethylation of H3K27Me3 and thus promotes gene expression. Here we identified Kdm6b as an important intracellular regulator of small intestinal IELs. Mice genetically deficient for Kdm6b showed greatly reduced numbers of TCRαβ^+^CD8αα^+^ IELs. In the absence of Kdm6b, TCRαβ^+^CD8αα^+^ IELs exhibited increased apoptosis, disturbed maturation and a compromised capability to lyse target cells. Both IL-15 and Kdm6b-mediated demethylation of histone 3 at lysine 27 are responsible for the maturation of TCRαβ^+^CD8αα^+^ IELs through upregulating the expression of Gzmb and Fasl. In addition, Kdm6b also regulates the expression of the gut-homing molecule CCR9 by controlling H3K27Me3 level at its promoter. However, Kdm6b is dispensable for the reactivity of thymic precursors of TCRαβ^+^CD8αα^+^ IELs (IELPs) to IL-15 and TGF-β. In conclusion, we showed that Kdm6b plays critical roles in the maturation and cytotoxic function of small intestinal TCRαβ^+^CD8αα^+^ IELs.

## Introduction

Intestinal intraepithelial lymphocytes (IELs) are mainly composed of conventional CD4^+^ and CD8αβ^+^ T cells and unconventional CD8αα^+^ T cells [1, 2]. Conventional IELs express αβ T cell receptor (TCR), while more than half of unconventional IELs express γδ TCR. Conventional IELs originate from peripheral naïve TCRαβ^+^CD4^+^ and TCRαβ^+^CD8αβ^+^ T cells which become antigen-primed lymphocytes in gut-associated lymphoid tissues or lymph nodes [3]. TCRαβ^+^CD8αα^+^ IELs derive from triple-positive (TP) thymic thymocytes (CD4^+^CD8α^+^CD8β^+^) that seed the intestinal epithelium as CD4^−^CD8α^−^CD8β^−^ precursor cells [4, 5]. After priming by non-self-antigens, conventional IELs upregulate the expression of α4β7, which interacts with mucosal addressin cell adhesion molecule 1 expressed by intestinal high endothelial venules, and CCR9, which can be recruited by CCL25 produced by intestinal epithelial cells (IECs). During the thymic stage of development, thymic precursors of TCRαβ^+^CD8αα^+^ IELs (IELPs) already express αEβ7 (αE is also known as CD103) and α4β7 [6]. The adhesion molecule αE integrin facilitates the localization of IELs to the intestinal epithelium by interacting with E-cadherin expressed by IECs [7–9]. Intestinal antigen-presenting cell- and IEC-derived IL-15 is required for the survival and further differentiation of IELs [10–13]. Nevertheless, the mechanisms regulating the development of intestinal IELs remain to be further investigated.

By survive negative selection in the thymus, TCRαβ^+^CD8αα^+^ IELs acquire an antigen-experienced phenotype and self-recognizing TCR repertoires [14, 15]. The lack of IL-13 from TCRγδ^+^CD8αα^+^ IELs increases the susceptibility of skin to cutaneous carcinogenesis [16]. The antitumor activity against colorectal cancer of human gut-resident NKp46-expressing intraepithelial Vδ1 T cells was also reported [17]. TCRαβ^+^CD8αα^+^ IELs express *IL-10*, *TGF-β3* and *Lag3*, implying their role in immune regulation. TCRαβ^+^CD8αα^+^ IELs inhibit the onset of colitis evoked by primary splenic TCRαβ^+^CD4^+^CD45RB^hi^ T cells in an IL-10 dependent manner [18, 19]. However, the cytotoxic function of TCRαβ^+^CD8αα^+^ IELs remains poorly understood.

The trimethylation of histone 3 at lysine 27 (H3K27Me3) is associated with gene repression [20, 21]. The JmjC catalytic domain-containing Kdm6 family proteins Kdm6a and Kdm6b are demethylases responsible for the demethylation of H3K27Me/2/3 [22]. Kdm6a negatively regulates the memory formation antigen-specific CD8^+^ T cells, and Kdm6b is essential for the differentiation of virus-specific CD8^+^ T cells and the generation of effector CD8^+^ T cells [23–25]. Kdm6b maintains Th17 cell differentiation and thymocyte egress by promoting the demethylation of H3K27Me3 at the *Rorc*, *S1pr1* and *Pdlim4* gene loci [26–28]. Abrogation of Kdm6b disturbs the lineage-specific epigenetic program of invariant natural killer T cells, which share a similar intrathymic development pathway to unconventional IELs [3, 29]. Kdm6b is upregulated during the effector response of mucosal-associated invariant T cells (a kind of unconventional T cells) [30]. Notably, vitamin D, which induces the expression of Kdm6b in colon cancer cells, is required for the development of IELs [31, 32]. However, whether and how Kdm6b regulates the development and function of IELs remain elusive.

Transcriptional factors, including T-bet, Runx3, Stat3, Smad3, cMyc and HIF-1, are important in the development, differentiation and function of IELs [33–37]. Nevertheless, the regulation of different IEL subsets, especially at the epigenetic level, remains poorly understood. In this study, we demonstrated that Kdm6b plays crucial roles in the development and function of small intestinal TCRαβ^+^CD8αα^+^ IELs. Loss of Kdm6b impairs IELPs homeostasis and migration and promotes TCRαβ^+^CD8αα^+^ IELs apoptosis, therefore leading to lower numbers of intestinal TCRαβ^+^CD8αα^+^ IELs. When crossed with *Apc*^Min/+^ mice, Kdm6b deficiency in IELs aggravated mutant-induced tumorigenesis in the small intestine. Mechanistically, Kdm6b promotes the expression of the antiapoptotic gene *Bcl2* and the cytotoxic gene *Gzmb* and *Fasl* in TCRαβ^+^CD8αα^+^ IELs through removal of the repressive marker H3K27Me3 in the enhancer and promoter, respectively. Collectively, we established Kdm6b as a previously unrecognized epigenetic regulator of IEL development and function.

## Results

### Kdm6b controls the heterogeneity and TCR repertoire of small intestinal IELs

**Fig. 1:**
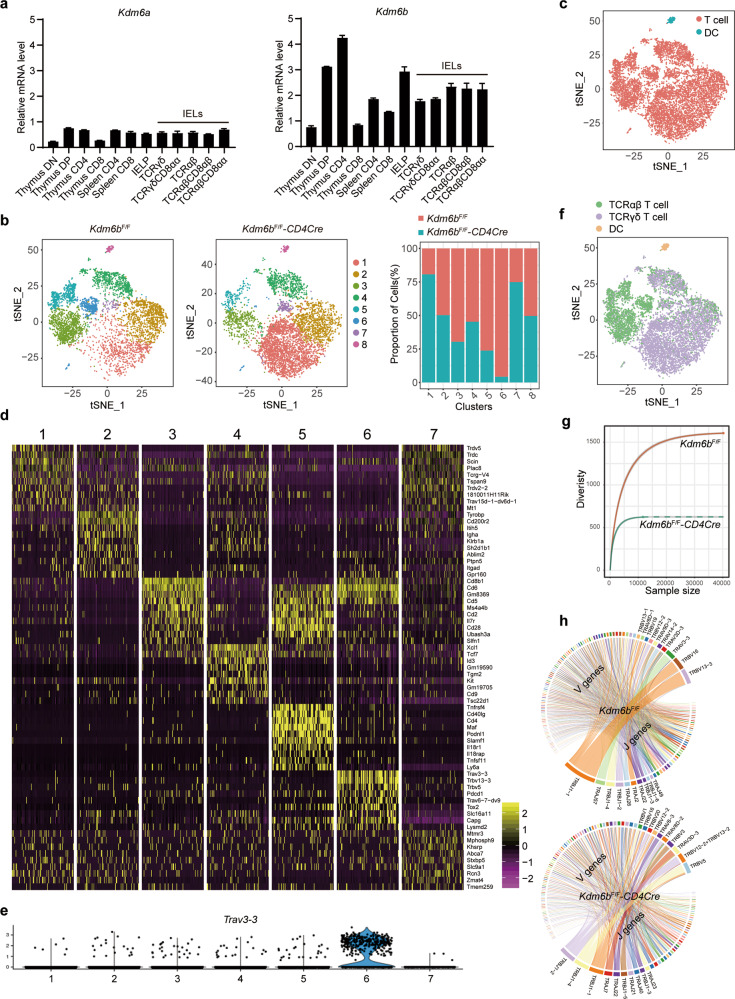
Deficiency of Kdm6b in T cells alters the heterogeneity and TCR repertoire of small intestinal IELs. **a** RT-qPCR analysis of the indicated genes using cDNA of indicated cells sorted from C57BL/6 mice. Results were depicted as n-fold difference relative to *Hprt1*. **b** t-distributed stochastic neighbor embedding (t-SNE) plots of scRNA-seq data of small intestinal CD45^+^ IELs from indicated mice showing clusters. Relative cell frequencies of each cluster are shown on the right. **c** Merged t-SNE plot of *Kdm6b*^F/F^-*CD4Cre* and *Kdm6b*^F/F^ mice showing the cell types among CD45^+^ IELs. **d** Heatmap of top marker genes of indicated clusters (from cluster 1 to 7). **e** Violin plots displaying the topmost marker gene of cluster 6. **f** Merged t-SNE plot of *Kdm6b*^F/F^-*CD4Cre* and *Kdm6b*^F/F^ mice displaying the expression of TCRαβ and TCRγδ in IELs at single-cell level. **g** Rarefaction curve showing the diversity of TCR repertoires in CD45^+^ IELs of *Kdm6b*^F/F^-*CD4Cre* and *Kdm6b*^F/F^ mice. **h** Circos plots showing the abundance of different V-J gene combinations in CD45^+^ IELs of *Kdm6b*^F/F^-*CD4Cre* and *Kdm6b*^F/F^ mice.

Among thymocytes (DN, DP, CD4^+^, and CD8^+^), spleen CD4^+^ and CD8^+^ T cells, thymic IELPs, and different small intestinal TCRαβ^+^ and TCRγδ^+^ IEL subsets (Fig. S1a, b), *Kdm6b* was expressed at higher levels than *Kdm6a* in different IEL subsets (Fig. 1a). Then we generated mice with T cell-specific deletion of *Kdm6b* by crossing mice harboring loxP-Flanked *Kdm6b* alleles (*Kdm6b*^F/F^) with *CD4Cre* transgenic mice expressing *Cre* recombinase starting from the late DN stage [28]. CD45^+^ small intestinal IELs were subjected to single-cell RNA-sequencing (scRNA-seq) (Fig. S1a). A total of 5880 cells from *Kdm6b*^F/F^ mice and 6488 cells from *Kdm6b*^F/F^-*CD4Cre* mice were sequenced. Cells were subjected to t-distributed stochastic neighbor embedding (t-SNE) analysis, and eight clusters in both groups were obtained (Fig. 1b). Cluster 1, 3, 5, 6 and 7 of the two groups had greatly different relative proportions, indicating that the deficiency of Kdm6b in T cells changed the heterogeneity of small intestinal IELs (Fig. 1b). All clusters were identified as T cells except cluster 8 which was defined as dendritic cells (Fig. 1c). Cluster 6 which displayed greatest reduction expressed top marker gene *Trav3–3* (Fig. 1d and 1e). By analyzing TCR expression, we revealed that the proportion of TCRαβ^+^ cells (including cluster 3, 5, and 6) was decreased and that the proportion of TCRγδ^+^ cells (including cluster 1 and 7) was increased in Kdm6b-deficient mice (Fig. 1f). The diversity of TCR repertoire was largely reduced in *Kdm6b*^F/F^-*CD4Cre* mice (Fig. 1g). Furthermore, the loss of Kdm6b resulted in very different TCR V-J gene combinations, with *Trbv13–3*-*Trbj1–1* as the most dominant V-J gene combination in Kdm6b-sufficient IELs and *Trbv5*-*Trbj1–4* as the most used V-J gene combination in kdm6b-deficient IELs (Fig. 1h). These results suggested pivotal roles of Kdm6b in the homeostasis of intestinal IELs.

### Kdm6b-deficiency reduces the number of TCRαβ^+^CD8αα^+^ IELs

**Fig. 2:**
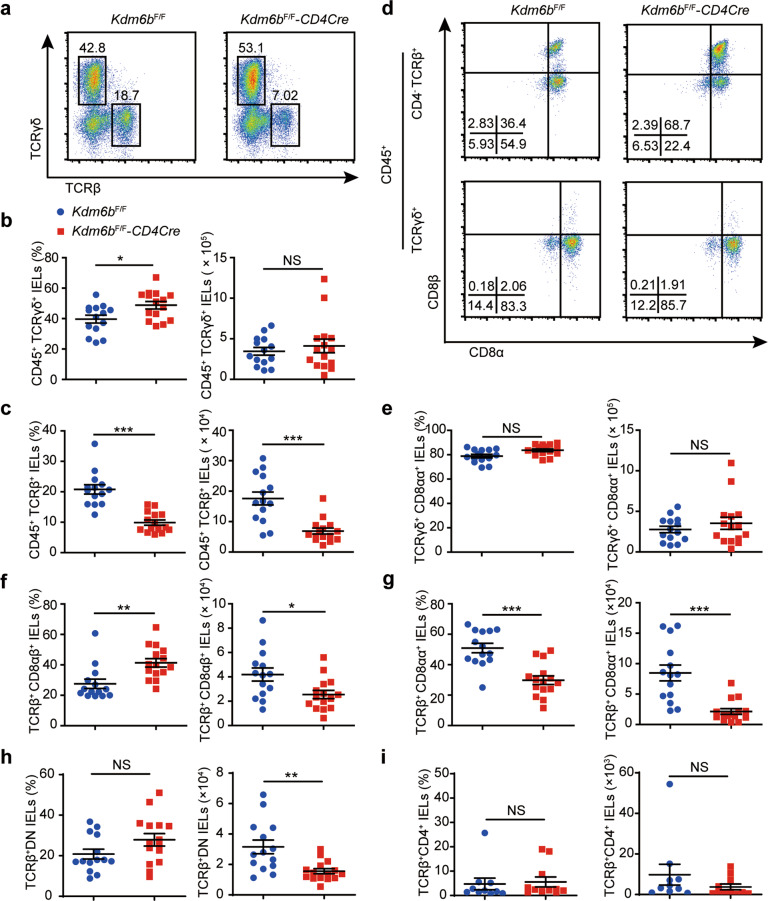
Deficiency of Kdm6b in T cells leads to the selective reduction of TCRαβ^+^ IEL subset in small intestine. **a** Flow cytometry analysis for the expression of TCRβ and TCRγδ in CD45^+^ IELs from the small intestines of indicated mice. **b, c** Percentages and absolute cell numbers of indicated subsets from *Kdm6b*^F/F^-*CD4Cre* and *Kdm6b*^F/F^ mice (*n* = 14–15). **d** Small intestinal IELs from *Kdm6b*^F/F^-*CD4Cre* and *Kdm6b*^F/F^ mice were stained with anti-CD45, TCRβ, TCRγδ, CD4, CD8α, and CD8β. The numbers in the dot plots represent the frequencies of cell in the quadrants. **e**–**i** Percentages and absolute cell numbers of indicated subsets from *Kdm6b*^F/F^-*CD4Cre* and *Kdm6b*^F/F^ mice (*n* = 14–15). Data in (**b**, **c**, **e**, **f**, **g**, **h** and **i**) are pooled from at least three independent experiments. Each symbol represents an individual mouse.

We further analyzed different IEL subsets by flow cytometry. The percentages and numbers of CD45^+^ small intestinal IELs were comparable between two group (Fig. S2a, b). Despite the increased frequencies of TCRγδ^+^ IELs, *Kdm6b*^F/F^-*CD4Cre* and control mice displayed similar numbers and frequencies of TCRγδ^+^CD88αα^+^ IELs (Fig. 2a, b, d, e). In contrast, the deletion of Kdm6b resulted in significantly reduced cell numbers and percentages of TCRαβ^+^ IELs, among which unconventional TCRαβ^+^CD8αα^+^ IELs displayed substantial reduction and conventional TCRαβ^+^CD8αβ^+^ IELs showed significant reduction but to a lesser extent (Fig. 2a, c, d, f, g). Interestingly, TCRαβ^+^DN (TCRαβ^+^CD8α^−^CD8β^−^) IELs, which represent the thymic precursors of TCRαβ^+^CD8αα^+^ IELs in the intestine, showed significant reduction in cell number by the absence of Kdm6b (Fig. 2h). The numbers and frequencies of TCRαβ^+^CD4^+^ IELs remain intact in Kdm6b-deficient mice (Fig. 2i). Taken together, these results indicated that the abnormal IELs heterogeneity in *Kdm6b*^F/F^-*CD4Cre* mice could be mainly attributed to reduced TCRαβ^+^ IELs and that Kdm6b played an important role in the regulation of unconventional TCRαβ^+^CD8αα^+^ IELs.

### Kdm6b is essential for maintaining the homeostasis of thymic IELPs

TCRαβ^+^CD8αα^+^ IELs arise from thymic IELPs (defined as CD4^−^CD8α^−^NK1.1^−^B220^-^TCRαβ^+^CD5^+^) (Fig. S1b). Compared with control group, the number of total thymocytes was similar in Kdm6b-deficient group (Fig. S3a), while the percentages and numbers of IELPs in Kdm6b-deficient group showed great increases relative to those in control group (Fig. S3b). The frequency of Ki67-positive thymic IELPs in *Kdm6b*^F/F^-*CD4Cre* mice was much lower than that in control mice (Fig. S3c). Staining with Annexin V/7-AAD revealed no difference in the cell death of IELPs between *Kdm6b*^F/F^-*CD4Cre* and *Kdm6b*^F/F^ mice (Fig. S3d). Consistently, the frequency of cleaved caspase 3-positive IELPs showed no difference between two groups (Fig. S3e). The antiapoptotic molecule Bcl2 was found to be similar between *Kdm6b*^*F/F*^-*CD4Cre* and *Kdm6b*^F/F^ mice (Fig. S3f, g). Thymic IELPs are further divided into PD-1^+^ and PD-1^−^ subsets [38]. We evaluated whether Kdm6b affected the expression of PD-1 and found both groups displayed equivalent percentages of PD-1^+^ IELPs (Fig. S3h). Thymocytes were labeled with FITC by intrathymic injection of FITC, and reduced FITC^+^ IELPs were detected in the small intestine and spleen of Kdm6b-deficient mice (Fig. S3i, j), consistent with fewer TCRαβ^+^DN IELs by the loss of Kdm6b (Fig. 2h). Altogether, these data suggested that Kdm6b plays a role in maintaining the homeostasis of thymic IELPs.

### Kdm6b is intrinsically required for the development of TCRαβ^+^CD8αα^+^ IELs

**Fig. 3:**
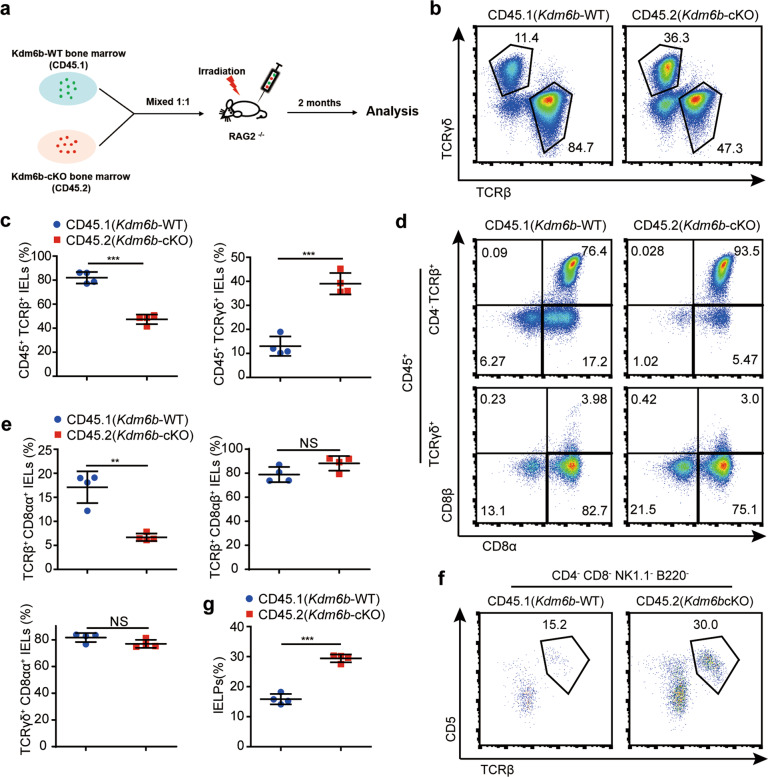
Kdm6b regulates TCRαβ^+^CD8αα^+^ IEL development in a cell-intrinsic manner. **a** 1:1 ratio of bone marrow cells (5 × 10^6^) from *Kdm6b*-cKO (CD45.2) and *Kdm6b*-WT (CD45.1) mice were transplanted into irradiated (at 600 rads) RAG2^−/−^ mice (on a CD45.1 background). Recipients were analyzed 8 weeks later. **b** Flow cytometry analysis for the expression of TCRβ and TCRγδ in CD45.1^+^ and CD45.2^+^ IELs from the small intestines of recipient mice. **c** Frequencies of TCRαβ^+^ and TCRγδ^+^ IELs from the experiment in (**b**) (*n* = 4). **d** Small intestinal IELs from recipient mice were stained with anti-CD45, TCRβ, TCRγδ, CD4, CD8α, and CD8β. The cells were evaluated by flow cytometry. **e** Percentages of indicated subsets from the experiment in (**d**) (*n* = 4). **f** IELPs in thymocytes of recipients were analyzed by flow cytometry. **g** Frequencies of CD45.1^+^ and CD45.2^+^ IELPs from the experiment in (**f**) (*n* = 4).

To explore whether the defects in TCRαβ^+^CD8αα^+^ IEL homeostasis induced by Kdm6b depletion are cell-intrinsic or just a secondary effect caused by the disturbance of other T cells. We generated mixed bone marrow (BM) chimaeras, in which conditional *Kdm6b*-KO and wild-type BM cells develop and differentiate in the same environment (Fig. 3a). The results revealed that Kdm6b was intrinsically required for the development of TCRαβ^+^ IELs, as demonstrated by the greatly decreased TCRαβ^+^ IELs in *Kdm6b*-cKO cells (Fig. 3b, c). The percentages of TCRαβ^+^CD8αα^+^ IELs from *Kdm6b*-cKO BM cells were reduced compared with those from wild-type BM cells (Fig. 3d, e). The frequencies of TCRγδ^+^CD88αα^+^ IELs from both *Kdm6b*-cKO and wild-type donors were comparably represented in chimaeras (Fig. 3d, e). The percentages of IELPs derived from *Kdm6b*-cKO BM cells were higher than those derived from wild-type BM cells, similar to the result in *Kdm6b*^F/F^-*CD4Cre* mice (Fig. 3f, g). These results demonstrated that Kdm6b plays a cell-intrinsic role in the development of TCRαβ^+^CD8αα^+^ IELs.

### Kdm6b is dispensable for IL-15- and TGF-β-induced maturation of IELPs

Treatment of thymic IELPs with IL-15 promotes the re-expression of CD8αα [12]. We surveyed the expression of IL-15 receptors, including CD215 (IL-15Rα), CD122(IL-15Rβ) and CD132 (γ_c_ chain), on TCRαβ^+^CD8αα^+^ IELs and IELPs. The results showed no difference between TCRαβ^+^CD8αα^+^ IELs of both groups (Fig. S4a, b). Their expression levels on thymic IELPs were also comparably represented (Fig. S4c, d). The IL-15 treatment of thymic IELPs from both *Kdm6b*^F/F^-*CD4Cre* and control mice exhibited equivalently enhanced levels of CD8αα re-expression (Fig. S4e). TGF-β controls the development of TCRαβ^+^CD8αα^+^ IELs by inducing the expression of CD8α in IELPs [35]. Both TCRαβ^+^CD8αα^+^ IELs and thymic IELPs from *Kdm6b*^F/F^-*CD4Cre* and *Kdm6b*^F/F^ mice shared comparable expression levels of *Tgfbr1* and *Tgfbr2* (Fig. S4f). Furthermore, thymic IELPs were treated with TGF-β, and their expression of *CD8a* was similar between the two groups (Fig. S4g). Thus, Kdm6b ablation does not affect the responsiveness of thymic IELPs to IL-15 and TGF-β treatment.

### Kdm6b regulates the gut homing of TCRαβ^+^CD8αα^+^ IELs

**Fig. 4:**
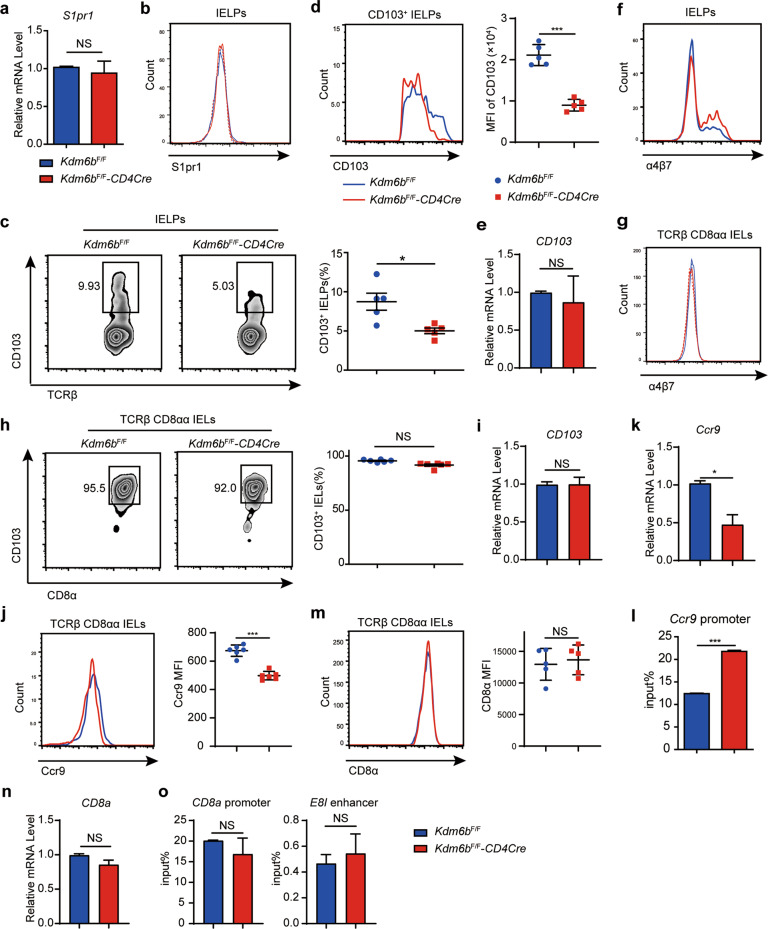
Kdm6b regulates the expression of molecules related to gut homing. **a** RT-qPCR analysis for the expression of *S1pr1* in the IELPs from *Kdm6b*^F/F^-*CD4Cre* and *Kdm6b*^F/F^ mice. **b** Flow cytometry analysis for the expression of S1pr1 in the IELPs from *Kdm6b*^F/F^-*CD4Cre* and *Kdm6b*^F/F^ mice (*n* = 5). **c** IELPs from *Kdm6b*^F/F^-*CD4Cre* and *Kdm6b*^F/F^ mice were stained with anti-CD103 and evaluated by flow cytometry. Statistical results are shown on the right (*n* = 5). **d** Geometric mean fluorescence intensity (MFI) of CD103 in CD103^+^ IELPs from indicated mice (*n* = 5). **e** RT-qPCR analysis for the expression of *CD103* in total IELPs of indicated mice. **f**, **g** IELPs and TCRαβ^+^CD8αα^+^ IELs from *Kdm6b*^F/F^-*CD4Cre* and *Kdm6b*^F/F^ mice were stained with anti-α4β7 and evaluated by flow cytometry. Representative result from three independent experiments were shown (*n* = 5). **h** TCRαβ^+^CD8αα^+^ IELs from *Kdm6b*^F/F^-*CD4Cre* and *Kdm6b*^F/F^ mice were stained with anti-CD103 and evaluated by flow cytometry. Statistical results are shown on the right (*n* = 6). **i** RT-qPCR analysis for the expression of *CD103* in TCRαβ^+^CD8αα^+^ IELs of indicated mice. **j** TCRαβ^+^CD8αα^+^ IELs from *Kdm6b*^F/F^-*CD4Cre* and *Kdm6b*^F/F^ mice were stained with anti-Ccr9 and evaluated by flow cytometry (*n* = 6). **k** RT-qPCR analysis for the expression of *Ccr9* in TCRαβ^+^CD8αα^+^ IELs of indicated mice. **l** ChIP-qPCR analysis of H3K27Me3 binding at the promoter of *Ccr9* in TCRαβ^+^CD8αα^+^ IELs from indicated mice. **m** Expression levels of CD8α were evaluated by flow cytometry (*n* = 5). **n** RT-qPCR analysis for the expression of *CD8a* in TCRαβ^+^CD8αα^+^ IELs of indicated mice. **o** ChIP-qPCR analysis of H3K27Me3 binding at the promoter and *E8I* enhancer of *CD8a* in TCRαβ^+^CD8αα^+^ IELs from indicated mice.

S1pr1 promotes thymocyte egress and is regulated by Kdm6b [27]. Consistently, we noted increased CD4^+^ T cells in the thymus and reduced CD4^+^ T cells in the peripheral lymph nodes and spleen of *Kdm6b*^F/F^-*CD4Cre* mice (Fig. S5a). Slightly reduced percentage and cell number of TCRαβ^+^ but not TCRγδ^+^ T cells in the spleen of *Kdm6b*^F/F^-*CD4Cre* mice was found (Fig. S5b, c, d). IELPs were tested for the expression level of S1pr1 and the results indicated Kdm6b was not required for its expression (Fig. 4a, b). α4β7, CCR9 and CD103 are critical gut homing receptors of IELs [6]. Although the α4β7^+^ IELPs in *Kdm6b*^F/F^-*CD4Cre* mice were not decreased (Fig. 4f), Kdm6b deficiency led to reduced frequencies of CD103^+^ IELPs and reduced MFI of CD103 in CD103^+^ IELPs (Fig. 4c, d). But the mRNA level of *CD103* remained unchanged in total IELPs of Kdm6b-deficient mice (Fig. 4e), in that Kdm6b might not regulate the mRNA level of *CD103* or the reduced *CD103* mRNA in very few CD103^+^ IELPs didn’t result in detectable alteration in total IELPs. The expression of α4β7 in TCRαβ^+^CD8αα^+^ IELs was not changed in the absence of Kdm6b (Fig. 4g). Almost all of TCRαβ^+^CD8αα^+^ IELs were CD103 positive, but their expression levels remained intact in *Kdm6b*^F/F^-*CD4Cre* mice (Fig. 4h, i). TCRαβ^+^CD8αα^+^ IELs of *Kdm6b*^F/F^-*CD4Cre* mice displayed lower CCR9 expression than *Kdm6b*^F/F^ mice (Fig. 4j, k). Chromatin immunoprecipitation-qPCR (ChIP-qPCR) experiments using TCRαβ^+^CD8αα^+^ IELs exhibited higher levels of H3K27Me3 at the promoter of *Ccr9* (Fig. 4l). The expression level of CD8α is similar between two groups (Fig. 4m, n). Consistently, H3K27Me3 binding at the promoter of *CD8a*, and the *E8I* enhancer, that controls the expression of *CD8a* in intestinal IELs [39, 40], showed no difference in TCRαβ^+^CD8αα^+^ IELs from *Kdm6b*^F/F^ and *Kdm6b*^F/F^-*CD4Cre* mice (Fig. 4o). Collectively, Kdm6b was required for IELPs to home to the intestinal epithelium in a CCR9- and CD103-dependent and α4β7-independent manner.

### Kdm6b controls the expression of TCRαβ^+^CD8αα^+^ IEL-specific genes

**Fig. 5:**
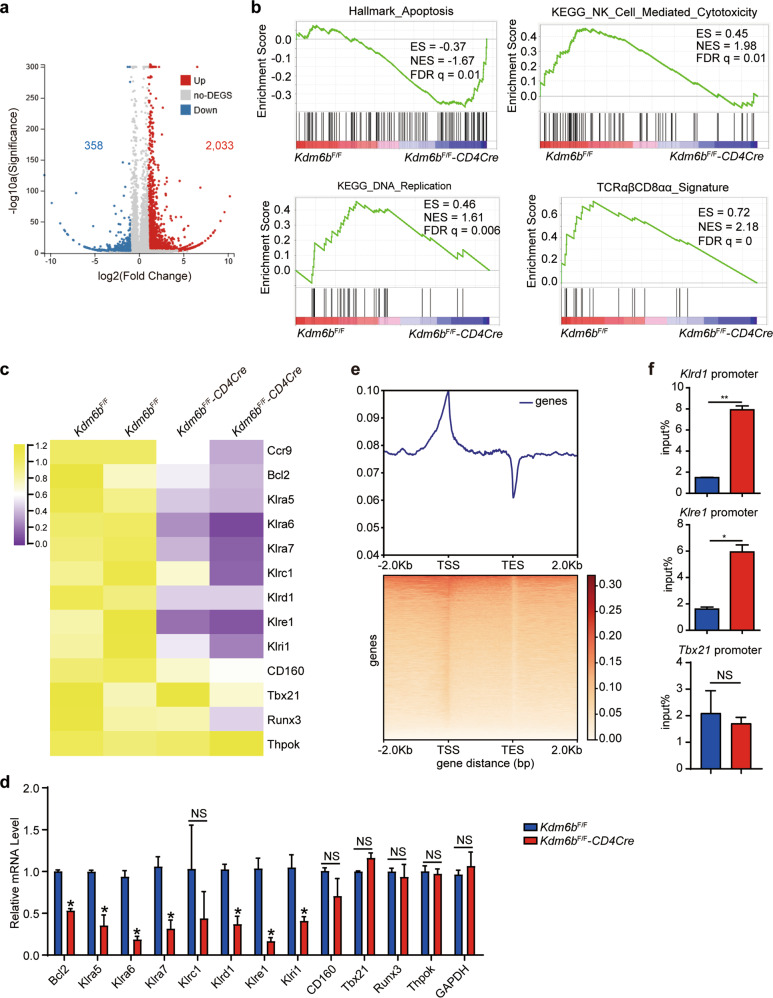
Loss of Kdm6b downregulates the expression of effector genes of TCRαβ^+^CD8αα^+^ IEL. **a** Scatter plots showing the differentially expressed genes identified by RNA-seq of TCRαβ^+^CD8αα^+^ IELs from *Kdm6b*^F/F^-*CD4Cre* and *Kdm6b*^F/F^ mice. Red and blue dots represent up- and downregulated genes in *Kdm6b*^F/F^-*CD4Cre* mice, respectively. **b** GSEA plots comparing TCRαβ^+^CD8αα^+^ IELs from *Kdm6b*^F/F^-*CD4Cre* and *Kdm6b*^F/F^ mice using indicated gene sets. Results are presented as enrichment score (ES), normalized enrichment score (NES) and false-discovery rate (FDR). **c** Heatmap showing the expression of selected genes in TCRαβ^+^CD8αα^+^ IELs from *Kdm6b*^F/F^-*CD4Cre* and *Kdm6b*^F/F^ mice. **d** RT-qPCR analysis of selected genes as in (**c**). **e** Heatmap of the distribution of H3K27Me3 decoration sites around transcription start site (TSS) and transcription end site (TES) of total genes detected through CUT&Tag-seq of TCRαβ^+^CD8αα^+^ IELs from wild-type C57BL/6 mice with anti-H3K27Me3. **f** ChIP-qPCR analysis of H3K27Me3 binding at the promoter of indicated genes in TCRαβ^+^CD8αα^+^ IELs from *Kdm6b*^F/F^-*CD4Cre* and *Kdm6b*^F/F^ mice.

Intestinal TCRαβ^+^CD8αα^+^ IELs were subjected to RNA sequencing. A total of 2391 genes were differentially expressed, with a log2 fold change > 1, by Kdm6b deficiency, among which 358 genes were downregulated and 2033 genes were upregulated (Fig. 5a). The differentially regulated genes were subjected to gene set enrichment analysis (GSEA) using the Molecular Signature Database gene sets and TCRαβ^+^CD8αα^+^ IEL-specific signature genes [18]. The results revealed enrichment in genes related to cell apoptosis, DNA replication and NK cell-mediated cytotoxicity (Fig. 5b). In addition, TCRαβ^+^CD8αα^+^ IEL-specific signature genes were also enriched (Fig. 5b). We mainly focused on the downregulated genes in *Kdm6b*^F/F^-*CD4Cre* mice (Fig. 5c). RT-qPCR verified some of the differentially expressed genes, including *Ccr9*, the antiapoptotic gene *Bcl-2*, and TCRαβ^+^CD8αα^+^ IEL-specific genes (*Klra5*, *Klra6*, *Klra7*, *Klrd1*, *Klre1*, and *Klri1*) (Figs. 4k and 5d). Transcriptional factors, including Tbx21 (also known as T-bet), Runx3 and Thpok, are involved in the regulation of intestinal IELs [33, 35]. In conventional CD8^+^ T cells, the expression of Tbx21, which directs the CD8^+^ lineage-specific transcriptional program, is controlled by H3K27Me3 [24, 41]. However, the expression of these transcriptional factors showed no alteration between two groups (Fig. 5c, d). Although identified by RNA-sequencing, some genes related to antiapoptotic signal (*Xiap*, *Mcl1*, and *Bclxl*) and DNA replication (*Mcm2*, *Mcm3*, *Mcm5*, *Rfc3*, *Lig1*, and *Pcna*) displayed no difference between *Kdm6b*^F/F^-*CD4Cre* and *Kdm6b*^F/F^ mice (Fig. S6a, b). The heatmap of H3K27Me3 CUT&Tag-seq with TCRαβ^+^CD8αα^+^ IELs revealed the enrichment of H3K27Me3 binding at the transcription start sites (TSS) at genomic level (Fig. 5e). ChIP-qPCR further exhibited higher levels of H3K27Me3 at the promoters of *Ccr9*, *Klrd1* and *Klre1* in *Kdm6b*^F/F^-*CD4Cre* mice than those in *Kdm6b*^F/F^ mice (Figs. 4l and 5f). These results implied Kdm6b participates in the regulation of apoptosis, cytotoxicity and the developmental program of TCRαβ^+^CD8αα^+^ IELs.

### Kdm6b protects TCRαβ^+^CD8αα^+^ IELs from apoptosis

**Fig. 6:**
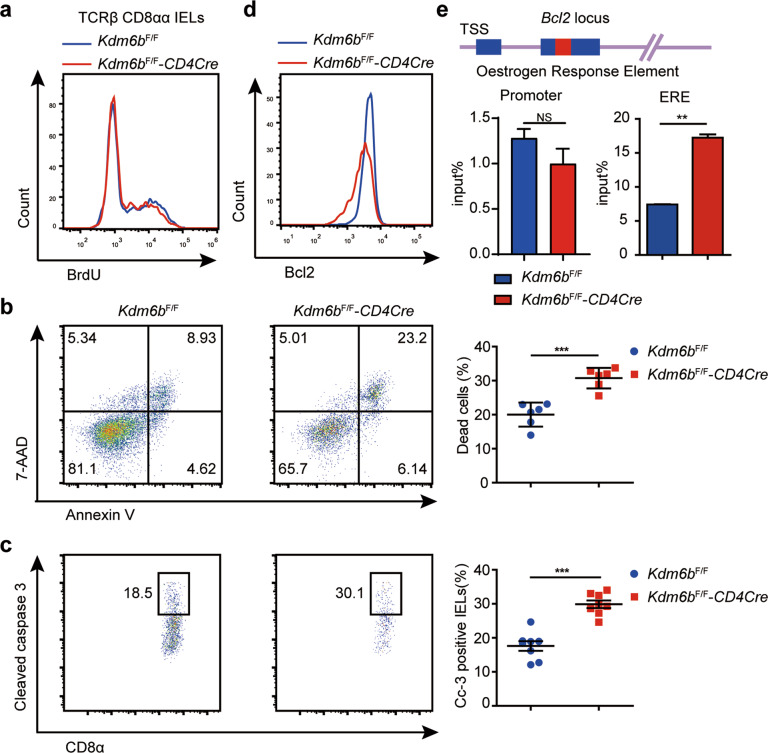
TCRαβ^+^CD8αα^+^ IEL displays increased apoptosis in Kdm6b-deficient mice. **a** *Kdm6b*^F/F^-*CD4Cre* and *Kdm6b*^F/F^ mice were injected intraperitoneally with BrdU (1.8 mg/mouse) and fed with BrdU in drinking water (0.8 mg/ml) for 2 days. BrdU incorporation in TCRαβ^+^CD8αα^+^ IELs were analyzed by flow cytometry (*n* = 5). **b** TCRαβ^+^CD8αα^+^ IELs from *Kdm6b*^F/F^-*CD4Cre* and *Kdm6b*^F/F^ mice were stained with Annexin V and 7-AAD and evaluated by flow cytometry. Representative dot plots and statistical results are shown (*n* = 6). **c** TCRαβ^+^CD8αα^+^ IELs from *Kdm6b*^F/F^-*CD4Cre* and *Kdm6b*^F/F^ mice were stained with anti-cleaved caspase 3 and evaluated by flow cytometry. Representative dot plots and statistical results are shown (*n* = 7). **d** Representative histogram showing comparison of Bcl2 in TCRαβ^+^CD8αα^+^ IELs from indicated mice as measured by flow cytometry (*n* = 6). **e** ChIP-qPCR analysis of H3K27Me3 binding at the promoter and ERE enhancer (Oestrogen Response Element) of *Bcl2* in TCRαβ^+^CD8αα^+^ IELs from *Kdm6b*^F/F^-*CD4Cre* and *Kdm6b*^F/F^ mice.

BrdU incorporation assays exhibited no difference between *Kdm6b*^F/F^-*CD4Cre* and control mice (Fig. 6a), consistent with our failure to verify the downregulated genes involved in DNA replication in *Kdm6b*^F/F^-*CD4Cre* mice (Fig. S6b). Dead cells were greatly increased in *Kdm6b*^F/F^-*CD4Cre* mice compared to control mice (Fig. 6b). Consistently, compared with those from *Kdm6b*^F/F^ mice, cleaved caspase 3 positive TCRαβ^+^CD8αα^+^ IELs from *Kdm6b*^F/F^-*CD4Cre* mice was apparently increased (Fig. 6c). Anti-Bcl2 antibody staining displayed lower Bcl2 intensity in the absence of Kdm6b (Fig. 6d). The expression of *Bcl2* was affected by an oestrogen response element existing in its second exon, which is decorated by H3K27Me3 in breast cancer cells [42]. ChIP-qPCR experiments using TCRαβ^+^CD8αα^+^ IELs revealed a higher level of H3K27Me3 at the oestrogen response element in *Kdm6b*^F/F^-*CD4Cre* mice than in *Kdm6b*^F/F^ mice, while their levels at *Bcl2* promoter remained the same (Fig. 6e). Taken together, above results suggested Kdm6b maintains the pool of TCRαβ^+^CD8αα^+^ IELs partly by promoting the expression of survival gene Bcl2.

### Loss of Kdm6b does not affect TCRαβ^+^CD8αα^+^ IELs-mediated immunosuppression and intestinal microbiota homeostasis

GSEA revealed enrichment for differentially expressed genes between regulatory and conventional T cells, implying an impaired immunoregulatory capability of Kdm6b-deficient TCRαβ^+^CD8αα^+^ IELs (Fig. S7a). DSS-provoked intestinal inflammation model showed that there was no difference in weight loss between *Kdm6b*^F/F^ and *Kdm6b*^F/F^-*CD4Cre* mice (Fig. S7b). RT-qPCR revealed similar levels of *IL-10* between Kdm6b-deficient and control TCRαβ^+^CD8αα^+^ IELs (Fig. S7c). We analyzed four major intestinal microorganism phyla, including Actinobacteria, Bacteroidetes, Firmicutes and Proteobacteria, and their representative classes, genera, or species using 16 S rDNA RT-qPCR. The relative constitution of intestinal bacteria remained intact in Kdm6b-deficient mice, regardless of whether the two groups of mice were housed in the same cage or separate cages (Fig. S7d–S7g). After oral infection with *Citrobacter rodentium* (*C. rodentium*), no significant difference was observed in bacterial burdens between *Kdm6b*^F/F^ and *Kdm6b*^F/F^-*CD4Cre* mice (Fig. S7h). These data suggested the immunoregulatory functions of intestinal IELs in intestinal inflammation and microbiota homeostasis might not be affected by the loss of Kdm6b.

### Loss of Kdm6b inhibits the maturation of TCRαβ^+^CD8αα^+^ IELs

**Fig. 7:**
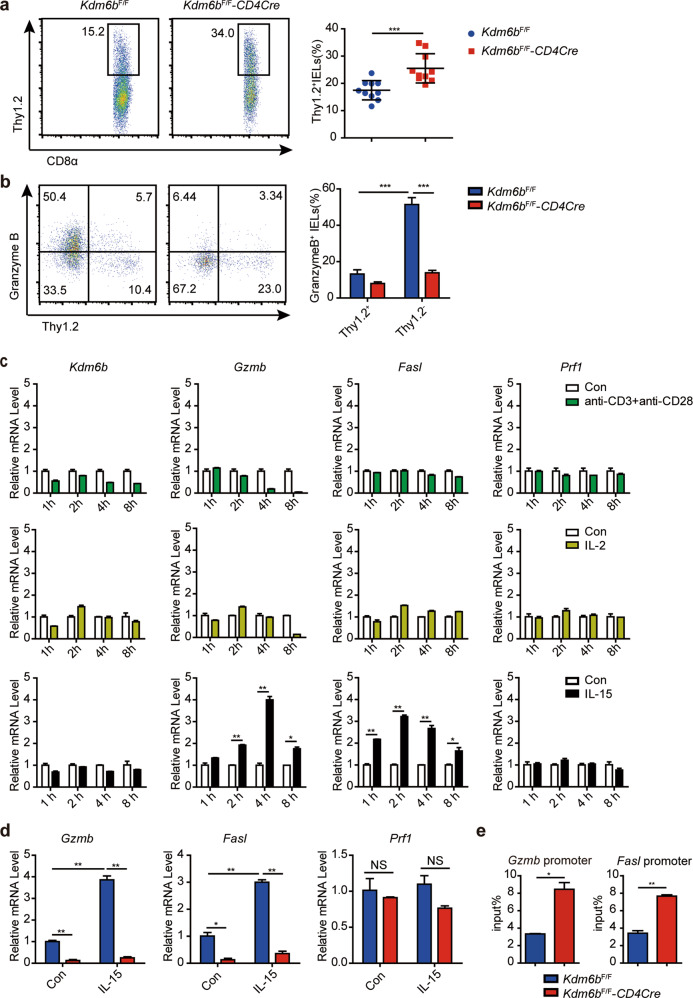
Kdm6b is required for the maturation of TCRαβ^+^CD8αα^+^ IEL. **a** TCRαβ^+^CD8αα^+^ IELs in *Kdm6b*^F/F^-*CD4Cre* and *Kdm6b*^F/F^ mice were analyzed for their expression of Thy1.2. Statistical results for the percentages of Thy1.2^+^TCRαβ^+^CD8αα^+^ IELs are shown on the right (*n* = 10). **b** TCRαβ^+^CD8αα^+^ IELs in *Kdm6b*^F/F^-*CD4Cre* and *Kdm6b*^F/F^ mice were analyzed for their expression of Thy1.2 and Gzmb. Statistical results are shown on the right (*n* = 6). **c** TCRαβ^+^CD8αα^+^ IELs of wild-type C57BL/6 mice were sorted and stimulated with anti-CD3 plus anti-CD28, IL-2 or IL-15. The expression of indicated genes were analyzed by RT-qPCR. **d** Sorted TCRαβ^+^CD8αα^+^ IELs from *Kdm6b*^F/F^-*CD4Cre* and *Kdm6b*^F/F^ mice were stimulated with IL-15 for 4 h and RT-qPCR was performed to detect the expression of indicated genes. **e** ChIP-qPCR analysis of H3K27Me3 binding at the promoter of indicated genes in TCRαβ^+^CD8αα^+^ IELs from *Kdm6b*^F/F^-*CD4Cre* and *Kdm6b*^F/F^ mice.

During their maturation, unconventional TCRαβ^+^CD8αα^+^ IELs lose Thy1.2 expression and acquire granzyme B (Gzmb) expression [34]. Compared with control mice, the frequency of Thy1.2^+^TCRαβ^+^CD8αα^+^ IELs in *Kdm6b*^F/F^-*CD4Cre* mice was significantly increased (Fig. 7a). The levels of Gzmb in Thy1.2^-^TCRαβ^+^CD8αα^+^ IELs exhibited great reduction in *Kdm6b*^F/F^-*CD4Cre* mice (Fig. 7b). IL-15, sharing two receptors with IL-2, supports the maturation of thymic IELPs and the expansion of cytotoxic T lymphocytes and enhances the antitumor properties of T cells [43]. We investigated the effect of IL-15, IL-2 and TCR stimulation on the expression of cytotoxic genes, and observed that IL-15, rather than IL-2 or TCR stimulation, upregulated the expression of *Gzmb* and *Fas ligand* (*Fasl*) in TCRαβ^+^CD8αα^+^ IELs (Fig. 7c). However, the expression of *Gzmb* and *Fasl* was significantly downregulated and could not be induced by IL-15 in Kdm6b-deficient TCRαβ^+^CD8αα^+^ IELs (Fig. 7d). None of above stimuli promoted the levels of *Kdm6b* and *Perforin* (*Prf1*) (Fig. 7c). Increased levels of H3K27Me3 at the promoter of *Gzmb* and *Fasl* were found through ChIP-qPCR experiments in TCRαβ^+^CD8αα^+^ IELs from *Kdm6b*^F/F^-*CD4Cre* mice (Fig. 7e). These results indicated that Kdm6b is essential for the maturation of TCRαβ^+^CD8αα^+^ IELs.

### Loss of Kdm6b suppresses the cytotoxic function of TCRαβ^+^CD8αα^+^ IELs

**Fig. 8:**
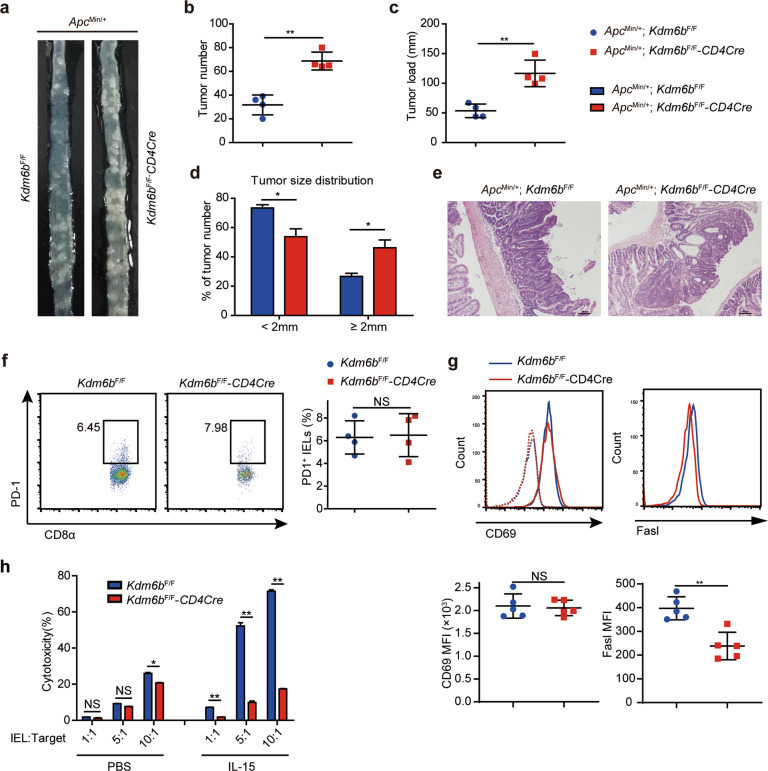
Loss of Kdm6b inhibits the cytotoxicity of TCRαβ^+^CD8αα^+^ IEL. **a**–**c** Macroscopic image (**a**), tumor number (**b**) and tumor load (**c**) of the small intestines of *Apc*^Min/+^; *Kdm6b*^F/F^-*CD4Cre* and *Apc*^Min/+^; *Kdm6b*^F/F^ mice at 14–16 weeks of age (*n* = 4). **d** Histogram showing the tumor size distribution of small intestines from indicated mice (*n* = 4). **e** H&E staining of the representative small intestines from indicted group of mice (*n* = 4). **f** TCRαβ^+^CD8αα^+^ IELs from *Kdm6b*^F/F^-*CD4Cre* and *Kdm6b*^F/F^ mice were stained with anti-PD-1 and evaluated by flow cytometry. Representative dot plots and statistical results are shown (*n* = 4). **g** Representative histogram showing comparison of CD69 and Fasl in TCRαβ^+^CD8αα^+^ IELs from *Kdm6b*^F/F^-*CD4Cre* and *Kdm6b*^F/F^ mice as measured by flow cytometry (*n* = 5). Dotted lines represent isotype controls. **h** Histogram showing the cytotoxicity of TCRαβ^+^CD8αα^+^ IELs from *Kdm6b*^F/F^-*CD4Cre* and *Kdm6b*^F/F^ mice against YAC-1 target cells at indicated cell ratios (IELs:YAC-1) with indicated stimuli.

Intestinal IELs display an “activated yet resting” and cytolytic status [44]. Compared with intestinal TCRαβ^+^CD8αβ^+^ IELs and TCRγδ^+^CD8αα^+^ IELs, TCRαβ^+^CD8αα^+^ IELs harbor a unique expression pattern of members of the Ly49 family of NK cell receptors, which are essential for cancer immunosurveillance mediated by NK cells [18, 45]. Intestinal IELs possess tumor cell-restricted spontaneous cytotoxicity [46]. We crossed *Kdm6b*^F/F^-*CD4Cre* mice with *Apc*^Min/+^ mice (an intestinal spontaneous adenomas mouse model driven by mutation of the WT *Apc* allele (*Apc*^Min/+^)) and analyzed the tumor progression. *Apc*^Min/+^; *Kdm6b*^F/F^-*CD4Cre* mice showed a significant increase in tumor number and tumor load compared with control mice (Fig. 8a, b, c). The tumors in compound *Apc*^Min/+^; *Kdm6b*^F/F^-*CD4Cre* mice were larger than those in control mice (Fig. 8d, e). To evaluate the tumor-killing activity of TCRαβ^+^CD8αα^+^ IELs, we detected the levels of PD-1, an inhibitory receptor of T cell cytotoxicity, and CD69, an activation marker of T cells, and found they remained similar levels between TCRαβ^+^CD8αα^+^ IELs from *Kdm6b*^F/F^ and *Kdm6b*^F/F^-*CD4Cre* mice (Fig. 8f, g). TCRαβ^+^CD8αα^+^ IELs in *Kdm6b*^F/F^-*CD4Cre* mice showed reduced expression of Fasl (Fig. 8g), which is related to the cytotoxic function of mature intestinal IELs [47]. TCRαβ^+^CD8αα^+^ IELs were co-cultured with YAC-1 target cells in vitro in the presence or absence of IL-15. We observed that TCRαβ^+^CD8αα^+^ IELs spontaneously lysed target cells in a cell number-dependent manner (Fig. 8h). Compared with IELs from *Kdm6b*^F/F^ mice, TCRαβ^+^CD8αα^+^ IELs from *Kdm6b*^F/F^-*CD4Cre* mice displayed compromised capability to lyse YAC-1 cells (Fig. 8h). In addition, IL-15 stimulation evidently promoted the cytotoxicity of TCRαβ^+^CD8αα^+^ IELs from Kdm6b-competent mice, but could not increase the ability of TCRαβ^+^CD8αα^+^ IELs from Kdm6b-deficient mice to lyse target cells (Fig. 8h). Collectively, these results suggested that Kdm6b-dependent IELs play a role in intestinal tumor surveillance and emphasized the important contribution of Kdm6b to the cytotoxicity of TCRαβ^+^CD8αα^+^ IELs.

## Discussion

The direct precursor of TCRαβ^+^CD8αα^+^ IEL comes from DN3 stage thymocytes, which undergo pre-TCR-driven β-selection and transition into the TP (CD4^+^CD8αβ^+^CD8αα^+^) stage. However, the precursors of intestinal TCRγδ^+^CD8αα^+^ IEL arise from multiple DN stages of thymocytes, among which γδ-lineage committed precursors directly home to epithelium after proceeding through γδ agonist selection without transition to the DP stage [4]. In the Kdm6b-deficient mice used in our research, the expression of *Cre* recombinase was mediated by the *CD4* gene promoter, so some precursors of TCRγδ^+^CD8αα^+^ IELs that originate from early DN stage cells do not express the *CD4* gene, thus, the expression of Kdm6b in these cells may not be affected in *Kdm6b*^F/F^-*CD4Cre* mice. This was confirmed by the normal expression of *Kdm6b* in TCRγδ IELs of *Kdm6b*^F/F^-*CD4Cre* mice (Fig. S8). Thus, the possibility that Kdm6b is a regulator of TCRγδ IELs cannot be excluded, which seems to be an interesting question worthy of further exploration using mice with Kdm6b deletion in early DN stage thymocytes. Despite the abnormal homeostasis of thymic IELPs, the increased IELP numbers and decreased Ki67-positive IELPs in Kdm6b-deficient mice seems contradictory, which is caused by the defects in IELP egress from the thymus. Nevertheless, the expression of S1pr1, a well-known gene responsible for thymocyte egress, was not altered in IELPs of *Kdm6b*^F/F^-*CD4Cre* mice. Therefore, the possibility that other genes regulated by Kdm6b in IELPs are responsible for thymic IELP egress exists and needs to be further investigated in the future.

An IL-10 dependent protective role of TCRαβ^+^CD8αα^+^ IELs was reported in the colitis of SCID mice induced by CD4^+^CD45RB^hi^TCRαβ^+^ thymus-derived T cells [19]. In contrast, another study showed that adoptive transfer of CD8α^+^ IELs into TCRβ×δ^-/-^ mice failed to confer protection against but rather aggravated the intestinal inflammation mediated by CD4^+^CD45RB^hi^ T cell. They displayed that TCRαβ^+^CD8αα^+^, TCRαβ^+^CD8αβ^+^ or TCRγδ^+^CD8αα^+^ IELs were unable to suppress the expansion of CD4^+^ T cells [48]. Although TCRαβ^+^CD8αα^+^ IELs are greatly reduced, DSS-induced body weight loss showed no difference between *Kdm6b*^F/F^ and *Kdm6b*^F/F^-*CD4Cre* mice. Thus, to some extent, our results suggested incapacity of intestinal TCRαβ^+^CD8αα^+^ IELs in immunosuppression of intestinal inflammation.

Despite their demethylase-independent activity, Kdm6a and Kdm6b are both responsible for the demethylation of H3K27Me3. They play complementary, counteractive or separate roles in the regulation of immune cells [21]. Kdm6a and Kdm6b synergistically regulates the differentiation of CD4^+^ T cell by promoting H3K27Me3 removal at, and expression of, *S1pr1* [27]. While they exhibited antagonistic activity in the development of acute T cell lymphoblastic leukemia [49, 50]. Kdm6a and Kdm6b can both control the M2 polarization of macrophage but in different demethylase-dependent manners [51, 52]. The level of Kdm6a in Kdm6b-deficient TCRαβ^+^CD8αα^+^ IELs remained the same as in control TCRαβ^+^CD8αα^+^ IELs (Fig. S9), suggesting the inexistence of compensation by Kdm6a for the loss of Kdm6b in TCRαβ^+^CD8αα^+^ IELs. Even though, the hypothesis that Kdm6a functions in the regulation of TCRαβ^+^CD8αα^+^ IELs cannot be negated, which needs to be further clarified using T cell conditional Kdm6a knockout mice in the future.

In the present study, Kdm6b was found to be essential for the survival, gut homing and function of small intestinal TCRαβ^+^CD8αα^+^ IELs. Notably, the tumor cell-restricted cytotoxicity of TCRαβ^+^CD8αα^+^ IELs was significantly attenuated by the loss of Kdm6b, thus resulting in defective immune surveillance in Kdm6b-deficient mice. Our results identified Kdm6b as a critical regulator of small intestinal TCRαβ^+^CD8αα^+^ IELs, and demonstrated that Kdm6b-deficient mice, which possessed significantly reduced mature intestinal TCRαβ^+^CD8αα^+^IELs and exhibited largely impaired cytotoxic function, developed *Apc*^*Min/+*^-driven intestinal adenomas at a higher malignant level. We showed that IL-15 is critical for IEL maturation and cytotoxicity, which requires Kdm6b-mediated upregulation of the expression of *Gzmb* and *Fasl*. In summary, Kdm6b-mediated H3K27Me3 demethylation in the context of IL-15 activation plays a critical role in maintaining the maturation and cytotoxic function of intestinal TCRαβ^+^CD8αα^+^ IELs.

## Materials and methods

### Mice

Mice were housed and bred at animal care facilities of Shanghai Institutes for Biological Sciences under specific pathogen-free conditions. *CD4*-*Cre* and loxP-Flanked *Kdm6b* mice have been described previously [28]. CD45.1 and RAG2^−/−^ mice are gifts from Dr. Ying Wang. The *Apc*^Min/+^ mice were from Model Animal Research Center of Nanjing University. All mice were maintained on the C57BL/6 background.

### Reagents

IL-2 (R&D, 402-ML), IL-15 (R&D, 447-ML), TGF-β1 (PeproTech, 100-21-10), HEPES (Gibco, 15630080), GlutaMAX^™^ Supplement (Gibco, 35050061), Sodium Pyruvate (Gibco, 11360070), β-Mercaptoethanol (Sigma, M3148), Non-Essential Amino Acids Solution (Gibco, 11140050), 7-AAD (BD Pharmingen^™^, 559925), Annexin V (BD Pharmingen^™^), CFSE (BD Pharmingen^™^, 565082), BrdU (Sigma, B5002), Formaldehyde (Sigma, 252549), Percoll (GE Healthcare Life Sciences, 17-0891-09).

### Antibodies

The following antibodies were used: Anti-CD3 (145-2C11, no azide and low endotoxin), anti-CD5 (53-7.3), anti-CD8α (53-6.7), anti-CD45.1 (A20), anti-CD45.2 (104), anti-CD69 (H1.2F3), anti-CD103 (M290), anti-TCRγδ (GL3), anti-NK1.1 (PK136), Thy1.2 (53-2.1), Anti-Fasl (MFL3), anti-α4β7 (DATK32) and anti-CD16/CD32 (2.4G2) were purchased from BD Biosciences. Anti-CD4 (GK1.5), anti-CD8β (H35-17.2), anti-CD45(30-F11), anti-CD122 (TM-b1 (TM-beta1)), anti-CD215 (DNT15Ra), anti-Granzyme B (NGZB), anti-TCRβ (H57-597), anti-Bcl2 (10C4), and anti-Ki67 (SolA15) were obtained from Invitrogen. Anti-CD132 (TUGm2), anti-CD45R/B220 (RA3-6B2), anti-PD-1 (29 F.1A12), and anti-CCR9 (9B1) were acquired from BioLegend. Anti-S1pr1 (R&D, 713412), anti-H3K27Me3 (Millipore, 07-449).

### Isolation of small intestinal IELs, thymocytes and splenocytes

For the isolation of IELs, mice were sacrificed and small intestines were removed and placed in ice-cold PBS. Residual mesenteric fat tissue and Peyer’s patches were carefully removed. Then the small intestines were opened longitudinally, washed thoroughly with PBS and cut into 0.5–1 cm pieces. The pieces were digested in HBSS containing 5% fetal bovine serum (FBS), 5 mM EDTA, 10 mM HEPES, 1 mM DTT at 37 °C with slow rotation for 20 min. After incubation the epithelial cell layer containing IELs was removed by intensive vortex for 10 s and passed through 100 μm cell strainer placed on a 50 mL Falcon tubes. The residual pieces were digested for a second time as described above. Pool of supernatants of both digestions was centrifuged and washed with PBS. The cell pellets were resuspended in 5 mL of 40% Percoll, overlaied on 5 mL of 80% Percoll in a 15 mL Falcon tube and centrifuged for 20 min at 1000 *g* at room temperature without brakes. After centrifuge, IELs were collected at the interphase of the two different Percoll solutions, washed twice with FACS buffer (PBS supplemented with 2% FBS) and used immediately for experiments. For the preparation of thymocytes and splenocytes, thymus and spleen were removed and mashed through a 70 μm cell strainer. Thymocytes and splenocytes were resuspended in FACS buffer and used for further experiments.

### Flow cytometry

For the staining of surface markers, cells were pre-incubated in FACS buffer containing anti-CD16/CD32 (1 μg/mL) for 15 min on ice to block Fc receptors, followed by incubation in FACS buffer with indicated fluorescent antibodies for 30 min on ice. For the detection of intracellular proteins, cells were fixed, permeabilized and stained with indicated antibodies with Foxp3/Transcription Factor Staining Buffer Set (eBioscience, 00-5523) according to the manufacturer’s instructions. The data were collected on a Gallios Flow Cytometer (Beckman Coulter) and analyzed using FlowJo (Tree Star) or Kaluza (Beckman Coulter) software.

### Mixed bone marrow chimeric mice

Bone marrow (BM) cells from the femurs and tibias of Kdm6b-sufficient (CD45.1^+^) mice and *Kdm6b*^F/F^-*CD4Cre* (CD45.2^+^) mice were isolated. And erythrocytes were lysed by incubation in ACK lysis buffer for 3 min. 2.5 × 10^6^ BM cells of Kdm6b-sufficient (CD45.1^+^) mice were mixed (1:1 ratio) with BM cells of Kdm6b^F/F^-CD4Cre (CD45.2^+^) mice and intravenously injected into recipient RAG2^−/−^ mice that had been irradiated at 600 rads once. Chimera were euthanized for further analysis after 8 weeks.

### In vitro cell culture

For IL-15 induction experiment, IELPs (CD4^−^CD8^−^B220^−^NK1.1^−^TCRβ^+^CD5^+^ thymocytes) were sorted and cultured in complete RPMI 1640 medium containing 10% FBS, 100 U/ml penicillin, 100 μg/ml streptomycin, 80 μM 2-mercaptoethanol, 10 mM HEPES, 8 mg/ml glutamine, nonessential amino acids and pyruvate in 96-well plates (2 × 10^4^ cells per well) supplemented with 100 ng/ml IL-15 for 1 week followed by flow cytometry. Sorted IELPs were cultured in complete RPMI 1640 medium supplemented with 2 ng/ml TGF-β1 in 96-well plates pre-coated with anti-CD3 overnight followed by RT-qPCR.

### RNA-seq

Small intestinal TCRαβ^+^CD8αα^+^IELs (CD45^+^CD4^−^TCRβ^+^CD8α^+^CD8β^−^) of control or *Kdm6b*^F/F^-*CD4Cre* mice were sorted into TRIzol Reagent (Life technologies, 15596-026) and total RNA was isolated according to manufacturer’s instructions. HiSeq RNA-Seq was performed in BGI Tech Solutions Co. Each sample contained pooled RNA from three mice with same genotype. Clean reads were mapped to the reference genome (mm10) using HISAT and Bowtie2. RSEM software package was used to quantify the level of gene expression.

### CUT&Tag-seq

Small intestinal TCRαβ^+^CD8αα^+^IELs were sorted from wild-type C57BL/6 mice and subject to CUT&Tag assay as previously described [53]. Anti-H3K27Me3 was used as the primary antibody to guide the proteinA/G-Tn5 transposon-mediated target DNA fragmentation. Purified DNA was used for library preparation using Hyperactive^TM^ In-Situ ChIP Library Prep Kit (TD901, Vazyme, China) and sequenced using PE150 by illumina Nova6000 sequencer. Clean reads were mapped to mm10 genome using Bowtie2. Peak calling was performed using SEACR software.

### scRNA-seq

Small intestinal CD45^+^ IELs were sorted and subject to scRNA-seq. Single cell suspensions were loaded onto the Chromium Single Cell Controller Instrument (10×Genomics, Pleasanton, CA, USA) to generate single cell gel beads in emulsions (GEMs). 5′ gene expression libraries and TCR libraries were constructed using a Chromium Single Cell 5′ Reagent Kit according to the manufacturer’s instructions. Full-length V(D)J segments were enriched from amplified cDNA with primers specific to TCR constant regions. All libraries were sequenced on the Illumina sequencing platform (HiSeq X Ten) with 150 bp paired-end read configuration. The Cell Ranger software pipeline (version 3.1.0) provided by 10×Genomics was used to demultiplex cellular barcodes, map reads to the genome and transcriptome using the STAR aligner, and down-sample reads as required to generate normalized aggregate data across samples, producing a matrix of gene counts versus cells. The unique molecular identifier (UMI) count matrix was processed by R package Seurat (version 3.0). We applied a criterion to filter out cells with UMI/gene numbers out of the limit of mean value ± 2 folds of standard deviations assuming a Guassian distribution of each cells’ UMI/gene numbers. Low-quality cells in which >10% of the counts belonged to mitochondrial genes were filtered out. We identified top variable genes across single cells and performed principal component analysis to reduce the dimensionality on the log transformed gene-barcode matrices of top variable genes. Cells were clustered according to a graph-based clustering approach and visualized in two-dimension using tSNE. Single-cell TCR data was processed using Cell Ranger with –reference=refdata-cellranger-vdj-GRCh38-alts-ensembl-3.1.0 to assemble TCR chains (V, (D), J and CDR3 nucleotide composition) and determine clonotypes for each sample.

### RT-qPCR

Total RNA of small intestinal IELs of control and *Kdm6b*^F/F^-*CD4Cre* mice are isolated as described above and converted to cDNA using PrimeScript^™^ RT reagent Kit with gDNA Eraser (TaKaRa, RR047A) according to manufacturer’s instructions. Real time PCR was conducted with SYBR^®^ Premix Ex Taq^™^ (TaKaRa, RR420A) on a Q7 RT-PCR detection system (Life technologies). The 2^−ΔΔCT^ cycle threshold method was used to calculate the relative change in expression. Results were normalized to the expression of Hprt1 mRNA. The primers used for RT-qPCR are listed in Supplementary Table 1.

### ChIP-qPCR

Sorted small intestinal TCRαβ^+^CD8αα^+^IELs (CD45^+^CD4^-^TCRβ^+^CD8α^+^CD8β^-^) were fixed with 1% formaldehyde and ChIP experiment was performed using Chromatin Immunoprecipitation Kit (Millipore, #17-371) according to manufacturer’s instructions. Anti-H3K27Me3 and anti-H3K4Me3 were used for immunoprecipitation. Purified ChIP DNA was quantified by qPCR with SYBR^®^ Premix Ex Taq^™^ and ChIP data were normalized to respective input sample. The primers used for RT-qPCR are listed in Supplementary Table 2.

### IELs cytotoxicity assay

Small intestinal TCRαβ^+^CD8αα^+^IELs (CD45^+^CD4^-^TCRβ^+^CD8α^+^CD8β^−^) of mice of indicated genotype were sorted and primed with PBS or IL-15 (100 ng/ml), and then co-cultured with CFSE-labeled target cells YAC-1 at different effector: target ratios (1:1, 5:1, 10:1) in complete RPMI 1640 medium. For the spontaneous death control, same number of CFSE-labeled YAC-1 was cultured alone in the same conditions. After 6 h of co-culture, cells were collected and labeled with 7-AAD. Lysed target cells were analyzed by flow cytometry and gated as CFSE^+^7-AAD^+^ cells.

### Intestinal microbiota analysis

Feces of mice of indicated genotype were collected and weighed. Small intestinal luminal feces were collected by flushing using sterile PBS and centrifuged at 6000 *g* for 10 min. The feces bacterial genomic DNA was isolated using E.Z.N.A.^®^ Stool DNA Kit (OMEGA, D4015-02) per manufacturer’s instructions. The stool genomic DNA was detected with SYBR^®^ Premix Ex Taq^™^ (TaKaRa, RR420A) on the Q7 RT-PCR detection system (Life technologies) to analyze the abundance of specific intestinal bacterial groups. Results were normalized to universal bacterial and the normalized CT value were used to calculate relative levels of 16 S rDNA gene expression of indicated bacterial groups. The 16 S rDNA primers used for qPCR analysis are listed in Supplementary Table 3.

### Statistics

All experiments were performed at least two independent times. Results in figures are pooled from independent experiments or represent one independent experiment with biological replicates (shown as mean ± SD). Sample size was determined based on the results of preliminary experiments. Statistical significance was calculated by two-tailed Student’s *t* test (*p < 0.05; **p < 0.01; ***p < 0.001; NS, not significant).

## Supplementary information

Supplementary Figure Legends

Supplementary Figure 1

Supplementary Figure 2

Supplementary Figure 3

Supplementary Figure 4

Supplementary Figure 5

Supplementary Figure 6

Supplementary Figure 7

Supplementary Figure 8

Supplementary Figure 9

Supplementary Table 1

Supplementary Table 2

Supplementary Table 3

## Data Availability

The GEO accession number of RNA-seq and CUT&Tag-seq reported in this paper is GSE154444 and GSE185798, respectively.
