# Supplementary material for: The histone demethylase Kdm6b regulates the maturation and cytotoxicity of TCRαβ+CD8αα+ intestinal intraepithelial lymphocytes
Source: Cell Death Differ. 2022 Jan 9;29(7):1349–63. doi: 10.1038/s41418-021-00921-w (PMC9287323; doi:10.1038/s41418-021-00921-w)
Supplement: Supplementary file 1 — Supplementary Figure Legends [file 41418_2021_921_MOESM1_ESM.docx]

**Supplementary** **figure legends**

**Supplementary Figure 1. Gating strategy for IEL and thymic IELP.**

**a,** TCRαβ^+^CD8αα^+^ IELs were gated as CD45^+^CD4^-^TCRβ^+^CD8α^+^CD8β^-^ cells. TCRαβ^+^CD8αβ^+^ IELs were gated as CD45^+^CD4^-^TCRβ^+^CD8α^+^CD8β^+^ cells. TCRγδ^+^CD8αα^+^ IELs were gated as CD45^+^CD4^-^TCRγδ^+^CD8α^+^CD8β^-^ cells. Subsets used for scRNA-seq were denoted. **b,** Thymic IELP was gated as CD4^-^CD8α^-^NK1.1^-^B220^-^TCRβ^+^CD5^+^ cells.

**Supplementary Figure 2. Proportion and number of CD45^+^ IEL is not affected by Kdm6b deficiency.**

**a,** Flow cytometry analysis for the CD45^+^ IELs from Percoll gradient-enriched cells of the small intestinal epithelium of *Kdm6b*^F/F^-*CD4Cre* and *Kdm6b*^F/F^ mice. **b,** Percentages and absolute cell numbers of CD45^+^ IELs are shown (n =14-15).

**Supplementary Figure 3. Kdm6b regulates the homeostasis of TCRαβ^+^CD8αα^+^ IEL precursor.**

**a,** Absolute numbers of thymocytes from *Kdm6b*^F/F^-*CD4Cre* and *Kdm6b*^F/F^ mice were counted (n =10). **b,** Thymocytes from *Kdm6b*^F/F^-*CD4Cre* and *Kdm6b*^F/F^ mice were stained with anti-CD4, CD8, NK1.1, B220, TCRβ, and CD5. Thymic IELPs were gated. Representative dot plots and statistical results are shown. Absolute numbers of IELPs were counted (n =10). **c,** IELPs of *Kdm6b*^F/F^-*CD4Cre* and *Kdm6b*^F/F^ mice were analyzed for the expression of Ki67. Representative dot plots and statistical results are shown (n =8-9). **d,** IELPs from *Kdm6b*^F/F^-*CD4Cre* and *Kdm6b*^F/F^ mice were stained with Annexin V and 7-AAD and evaluated by flow cytometry. Representative dot plots and statistical results are shown (n = 4). **e,** IELPs from *Kdm6b*^F/F^-*CD4Cre* and *Kdm6b*^F/F^ mice were stained with indicated antibodies and evaluated by flow cytometry. Representative dot plots and statistical results are shown (n = 4-5). **f,** RT-qPCR analysis of *Bcl2* using cDNA of IELPs from *Kdm6b*^F/F^-*CD4Cre* and *Kdm6b*^F/F^ mice. **g,** Representative histogram showing comparison of Bcl2 protein in IELPs from *Kdm6b*^F/F^-*CD4Cre* and *Kdm6b*^F/F^ mice as measured by flow cytometry (n = 4). **h,** IELPs from *Kdm6b*^F/F^-*CD4Cre* and *Kdm6b*^F/F^ mice were stained with anti-PD-1 and evaluated by flow cytometry. Representative dot plots and statistical results are shown (n = 5). **i,** FITC labeled IELPs of small intestine and spleen from *Kdm6b*^F/F^-*CD4Cre* and *Kdm6b*^F/F^ mice were analyzed by flow cytometry 24h after intrathymic injection of FITC (10μg/mouse). **j,** Statistical results of frequencies of FITC labeled IELPs as in (**i**) (n = 4).

**Supplementary Figure 4. Kdm6b-deficient IEL precursor remains normal reactivity to IL-15 and TGF-β.**

**a,** RT-qPCR analysis for the expression of indicated genes using cDNA of TCRαβ^+^CD8αα^+^ IELs sorted from *Kdm6b*^F/F^-*CD4Cre* and *Kdm6b*^F/F^ mice. **b,** Small intestinal TCRαβ^+^CD8αα^+^ IELs from *Kdm6b*^F/F^-*CD4Cre* and *Kdm6b*^F/F^ mice were stained with anti-CD215, CD122 and CD132. The cells were tested through flow cytometry (n = 6). **c,** RT-qPCR analysis for the expression of indicated genes using cDNA of IELPs sorted from *Kdm6b*^F/F^-*CD4Cre* and *Kdm6b*^F/F^ mice. **d,** IELPs from *Kdm6b*^F/F^-*CD4Cre* and *Kdm6b*^F/F^ mice were stained with anti-CD215, CD122 and CD132. The cells were tested through flow cytometry (n = 6). **e,** IELPs from thymocytes of *Kdm6b*^F/F^-*CD4Cre* and *Kdm6b*^F/F^ mice were sorted and cultured in the presence of IL-15. The cells were collected and analyzed for their expression of CD8α and CD8β by flow cytometry. Statistical results on right panel represent one of 3 independent experiments. **f,** RT-qPCR analysis for the expression of TGF-β receptors using cDNA of IELPs (left) and TCRαβ^+^CD8αα^+^ IELs (right) sorted from *Kdm6b*^F/F^-*CD4Cre* and *Kdm6b*^F/F^ mice. **g,** IELPs from *Kdm6b*^F/F^-*CD4Cre* and *Kdm6b*^F/F^ mice were sorted and cultured in the presence of TGF-β. The cells were collected and analyzed for their expression of *CD8a* by RT-qPCR.

**Supplementary Figure 5. Kdm6b regulates thymocyte egress.**

**a,** Thymus, spleen, mesenteric and inguinal lymph nodes of *Kdm6b*^F/F^-*CD4Cre* and *Kdm6b*^F/F^ mice were grinded and passed through cell strainer. The cells were stained with anti-CD4 and CD8 and evaluated by flow cytometry. Representative dot plots are shown (n = 5). **b,** Splenocytes were analyzed for their expression of TCRβ and TCRγδ. Representative dot plots are shown. **c, d,** Statistical results of the proportion (**c**) and cell number (**d**) of spleen TCRβ^+^ and TCRγδ^+^ cells as in (**b**) (n = 8).

**Supplementary Figure 6. Kdm6b is not required for the expression of genes related to DNA replication.**

**a, b,** RT-qPCR analysis verifying downregulated genes related with apoptosis (**a**) and DNA replication (**b**) identified by GSEA.

**Supplementary Figure 7. Kdm6b is not essential for TCRαβ^+^CD8αα^+^ IELs-mediated immunoregulation and intestinal microbiota homeostasis.**

**a,** GSEA plots comparing TCRαβ^+^CD8αα^+^ IELs from *Kdm6b*^F/F^-*CD4Cre* and *Kdm6b*^F/F^ mice using gene set of immunologic Treg VS Tcon. Result is presented as enrichment score (ES), normalized enrichment score (NES) and false-discovery rate (FDR). **b,** *Kdm6b*^F/F^-*CD4Cre* and *Kdm6b*^F/F^ mice were treated with 3% DSS in drinking water for 5 days, then replaced with normal drinking water. Body weights were recorded daily. Weight curves were given as percentage of initial body weight (n = 5). **c,** RT-qPCR analysis for the expression of *IL-10* using cDNA of TCRαβ^+^CD8αα^+^ IELs sorted from *Kdm6b*^F/F^-*CD4Cre* and *Kdm6b*^F/F^ mice. **d,** RT-qPCR analysis of 16S rDNA of Actinobacteria phylum, Bacteroidetes phylum, Firmicutes phylum and Proteobacteria phylum, and their representative classes, genera, or species in the small intestinal contents of *Kdm6b*^F/F^-*CD4Cre* and *Kdm6b*^F/F^ mice (n = 6). **e, f, g,** RT-qPCR analysis of 16S rDNA of Actinobacteria phylum, Bacteroidetes phylum, Firmicutes phylum and Proteobacteria phylum, and their representative classes, genera, or species in the feces of *Kdm6b*^F/F^-*CD4Cre* and *Kdm6b*^F/F^ mice housed in separated cages at day0 (**e**), day10 (**f**) and day30 (**g**) after separation (n = 6). **h,** *Kdm6b*^F/F^-*CD4Cre* and *Kdm6b*^F/F^ mice were infected with 10^9^ colony-forming unit (CFU) of *C. rodentium* by oral gavage. Feces were collected at indicated time points after infection. CFU counts of *C. rodentium* in the feces are shown (n = 6).

**Supplementary Figure 8. *CD4Cre* is unable to mediate the knockout of Kdm6b in TCRγδ^+^ IEL.**

RT-qPCR analysis for the expression of *Kdm6b* using cDNA of TCRαβ^+^ and TCRγδ^+^ IELs sorted from *Kdm6b*^F/F^-*CD4Cre* and *Kdm6b*^F/F^ mice.

**Supplementary Figure 9. The expression level of Kdm6a in Kdm6b-deficient TCRαβ^+^CD8αα^+^ IEL.**

RT-qPCR analysis for the expression of *Kdm6a* using cDNA of TCRαβ^+^CD8αα^+^ IELs sorted from *Kdm6b*^F/F^-*CD4Cre* and *Kdm6b*^F/F^ mice.
