# Supplementary figures and images for: The histone demethylase Kdm6b regulates the maturation and cytotoxicity of TCRαβ+CD8αα+ intestinal intraepithelial lymphocytes
Source: Cell Death Differ. 2022 Jan 9;29(7):1349–63. doi: 10.1038/s41418-021-00921-w (PMC9287323; doi:10.1038/s41418-021-00921-w)

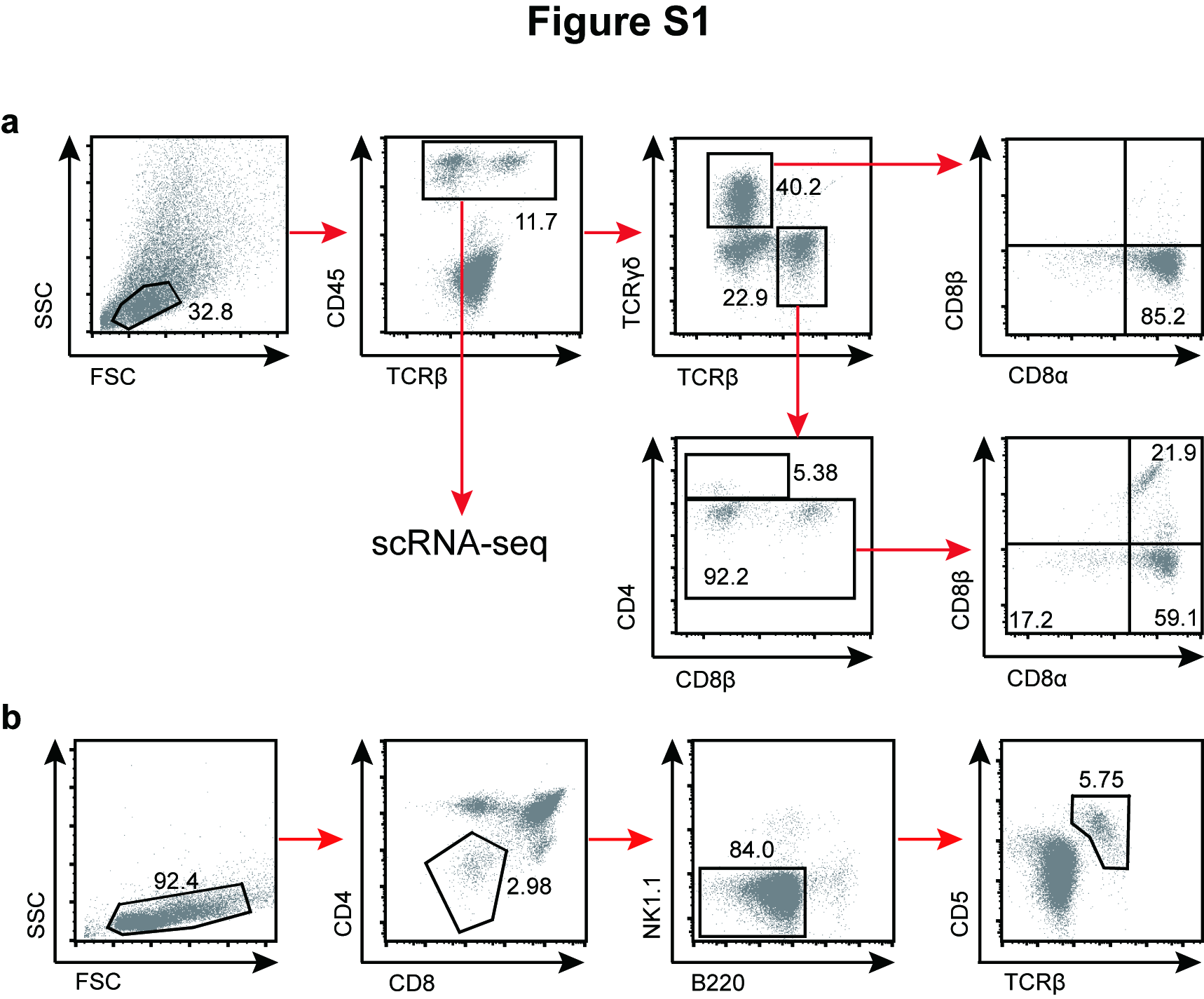

Supplement: Supplementary file 2 — Supplementary Figure 1 [file 41418_2021_921_MOESM2_ESM.tif]

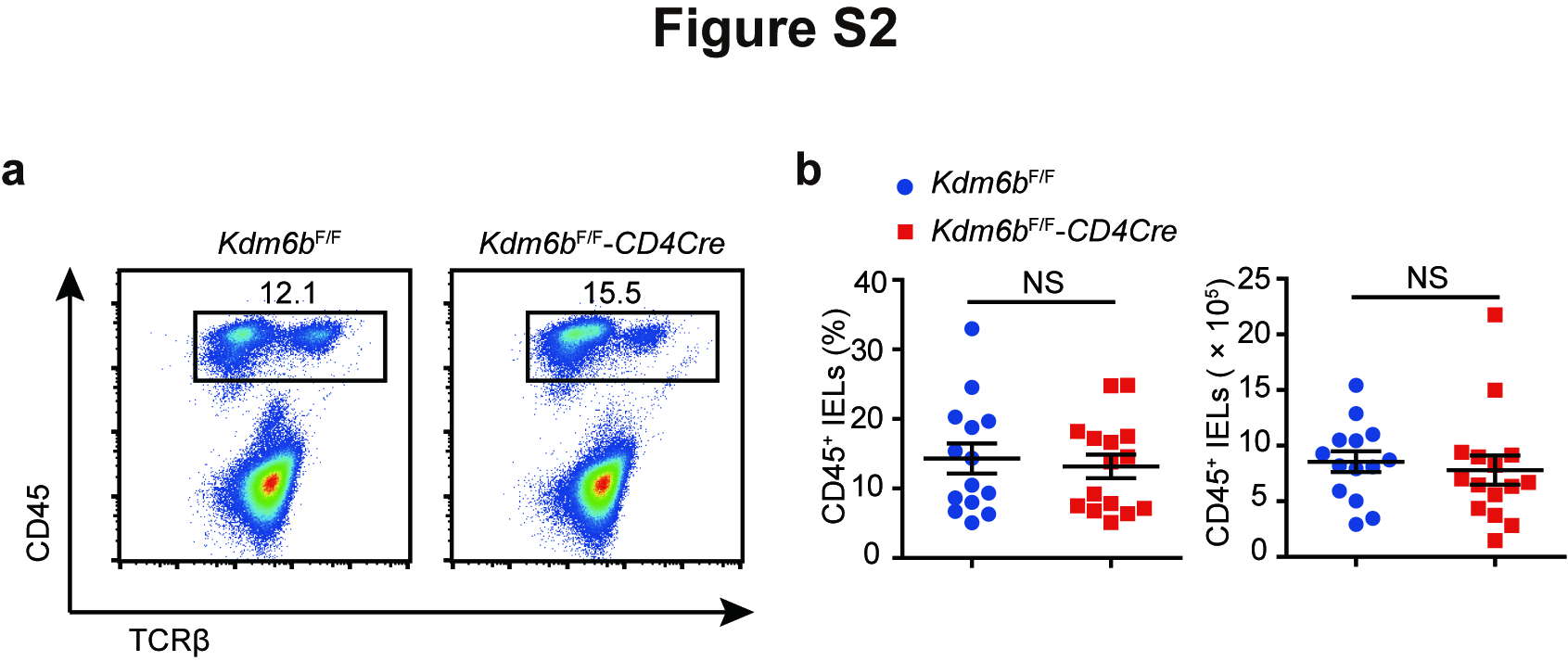

Supplement: Supplementary file 3 — Supplementary Figure 2 [file 41418_2021_921_MOESM3_ESM.tif]

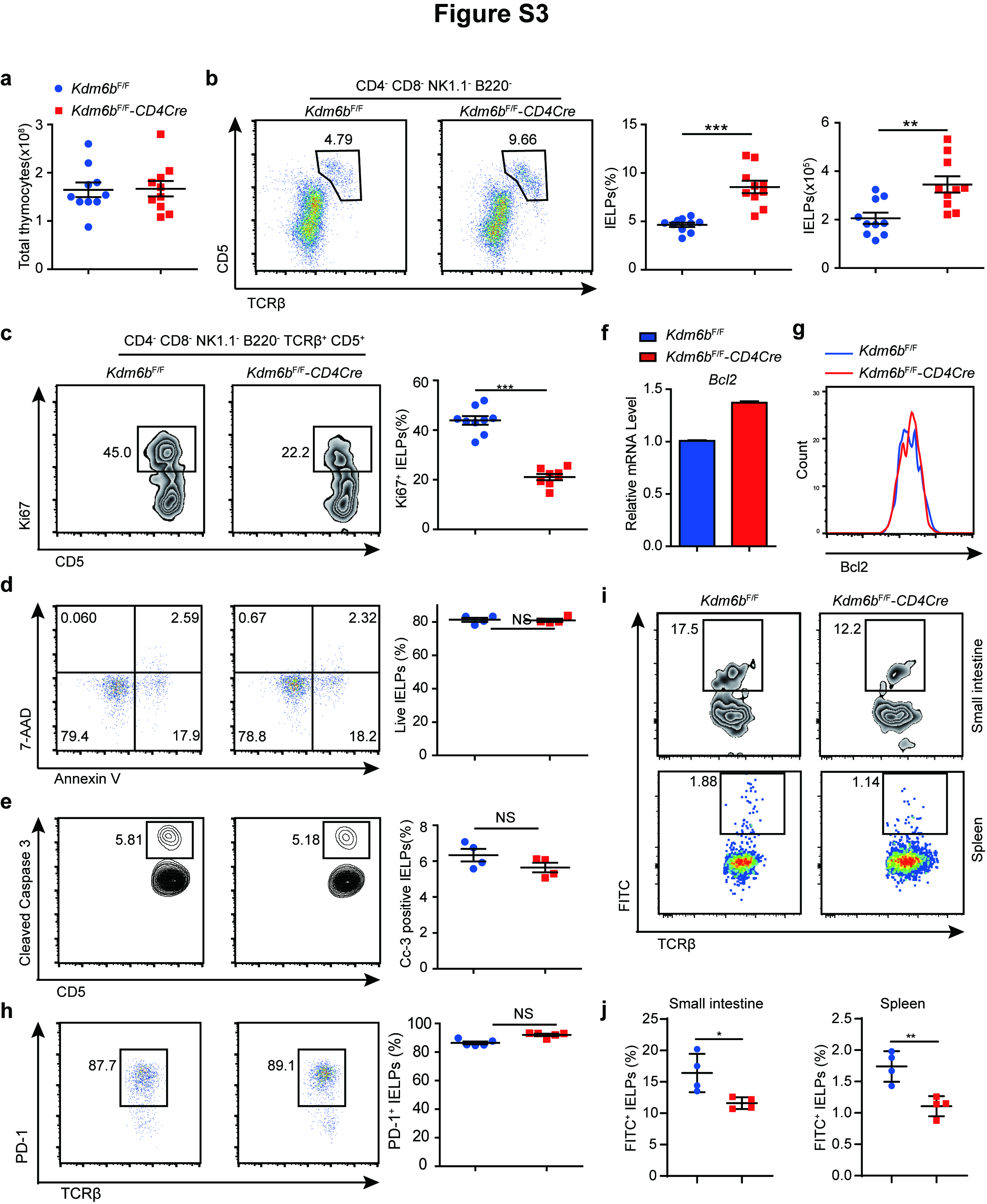

Supplement: Supplementary file 4 — Supplementary Figure 3 [file 41418_2021_921_MOESM4_ESM.tif]

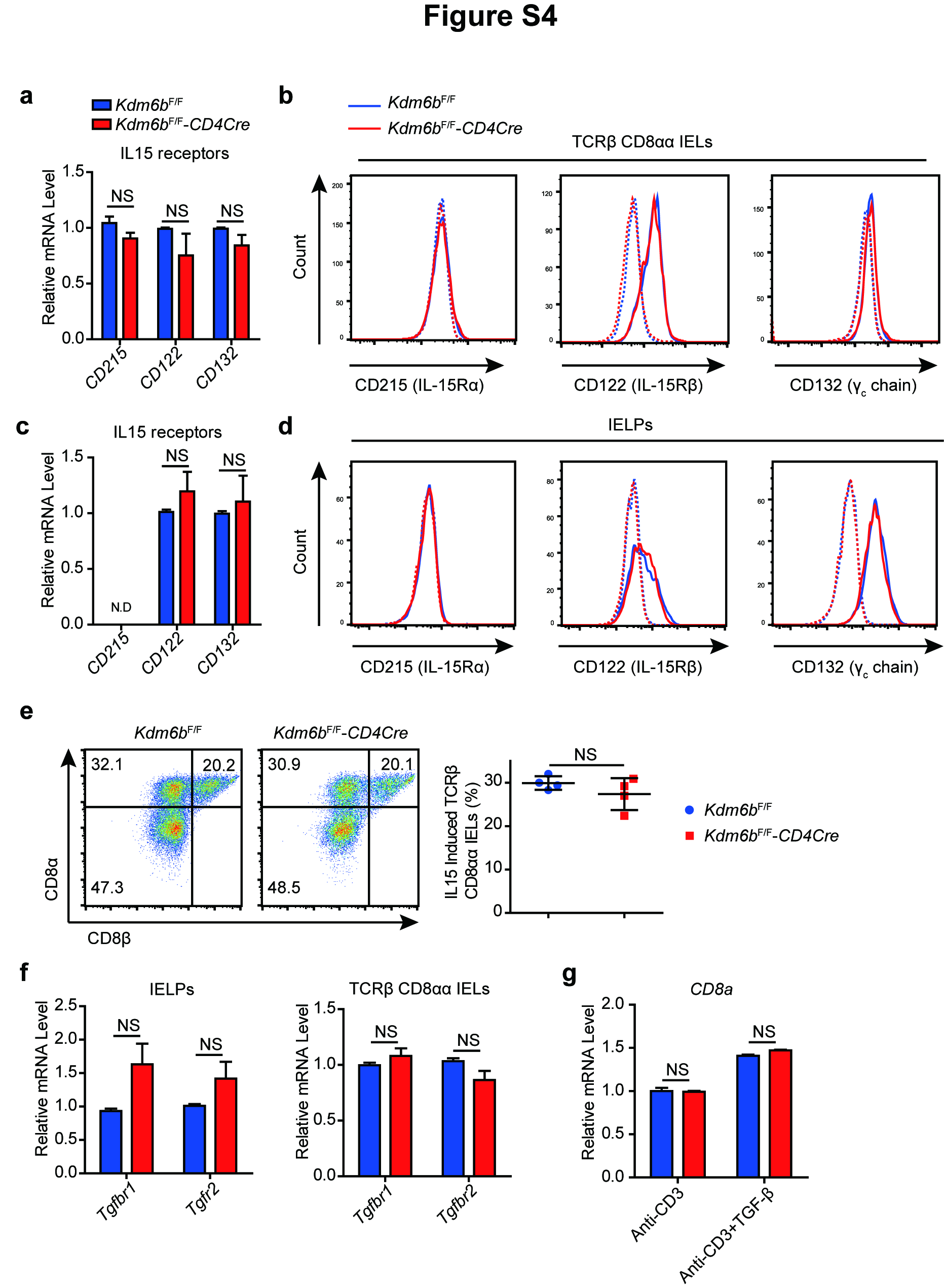

Supplement: Supplementary file 5 — Supplementary Figure 4 [file 41418_2021_921_MOESM5_ESM.tif]

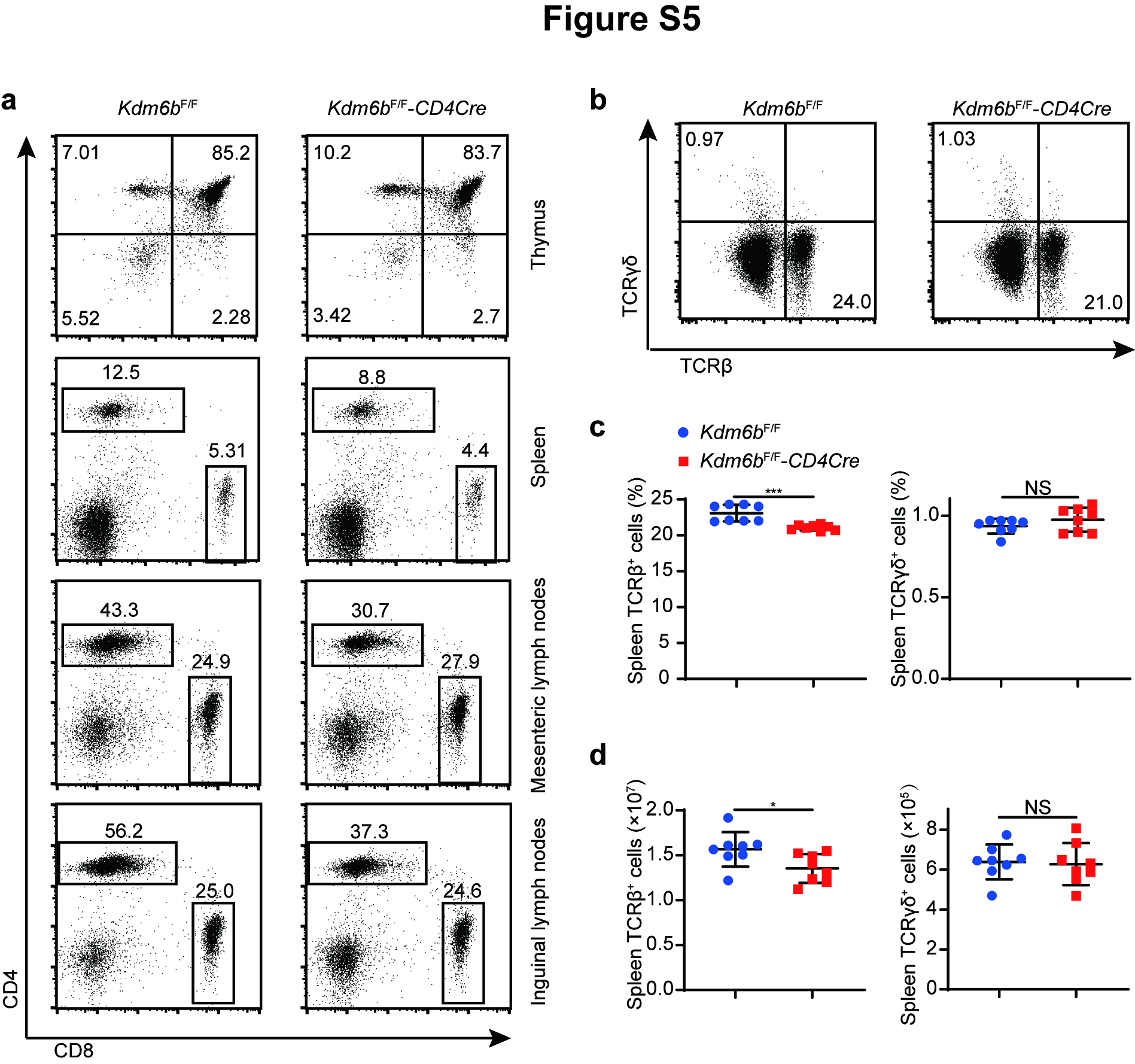

Supplement: Supplementary file 6 — Supplementary Figure 5 [file 41418_2021_921_MOESM6_ESM.tif]

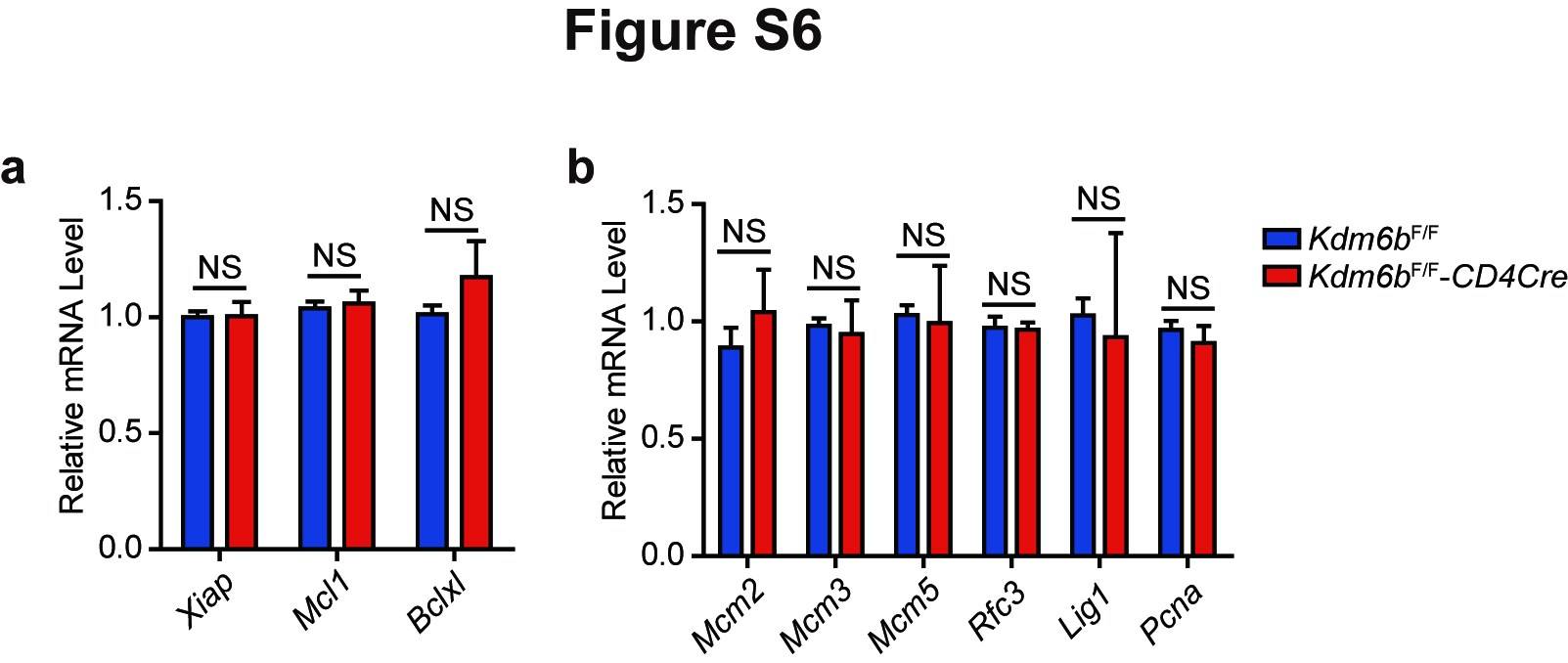

Supplement: Supplementary file 7 — Supplementary Figure 6 [file 41418_2021_921_MOESM7_ESM.tif]

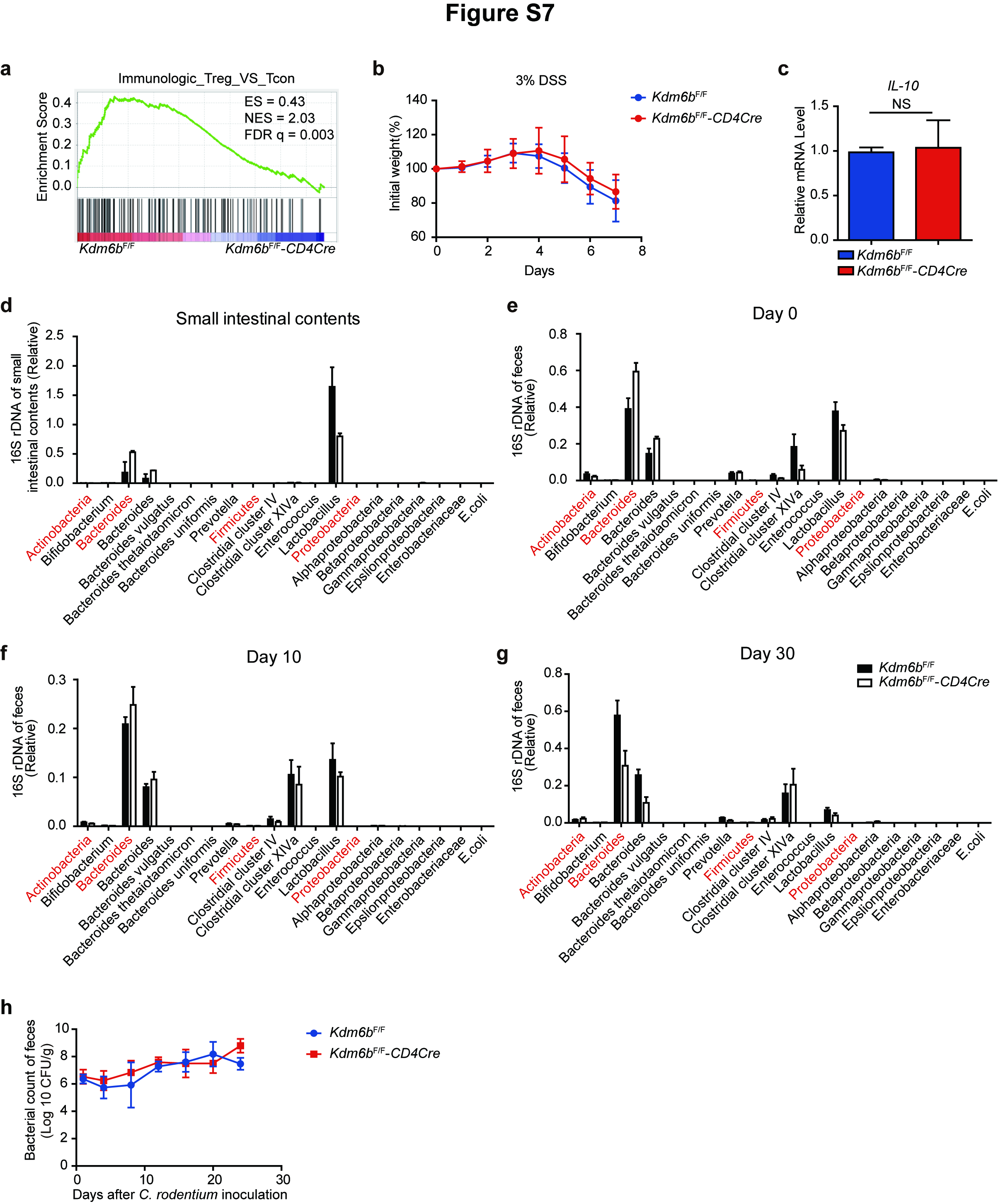

Supplement: Supplementary file 8 — Supplementary Figure 7 [file 41418_2021_921_MOESM8_ESM.tif]

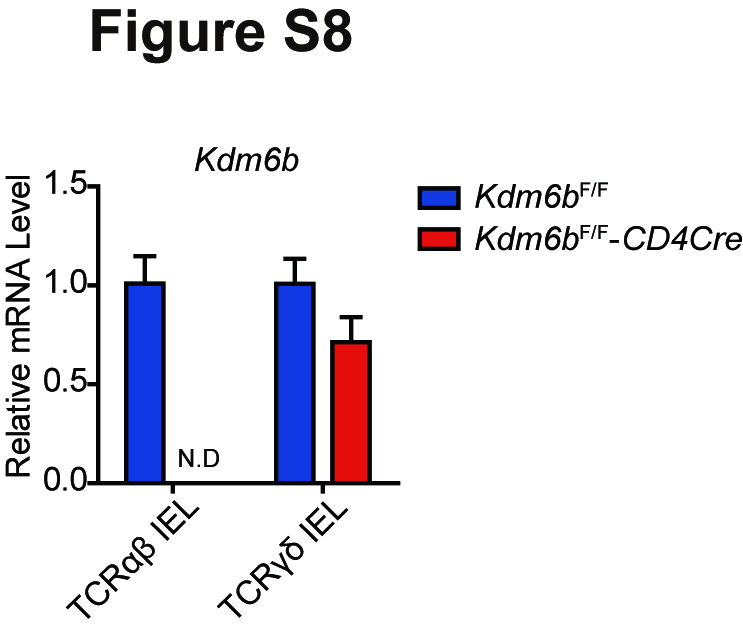

Supplement: Supplementary file 9 — Supplementary Figure 8 [file 41418_2021_921_MOESM9_ESM.tif]

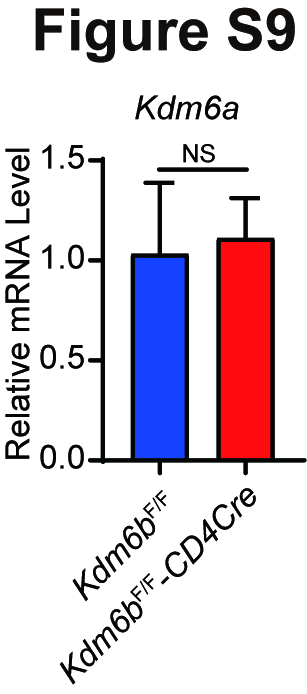

Supplement: Supplementary file 10 — Supplementary Figure 9 [file 41418_2021_921_MOESM10_ESM.tif]
