# Supplementary material for: The histone demethylase Kdm6b regulates the maturation and cytotoxicity of TCRαβ+CD8αα+ intestinal intraepithelial lymphocytes
Source: Cell Death Differ. 2022 Jan 9;29(7):1349–63. doi: 10.1038/s41418-021-00921-w (PMC9287323; doi:10.1038/s41418-021-00921-w)
Supplement: Supplementary file 11 — Supplementary Table 1 [file 41418_2021_921_MOESM11_ESM.docx]

| Supplementary Table 1. Primers used for RT-qPCR | |
| --- | --- |
| Gene name | Sequence |
| *Hprt1*-F | 5’-GCGTCGTGATTAGCGATGATG-3’ |
| *Hprt1*-R | 5’-CGAGCAAGTCTTTCAGTCCTGTCC-3’ |
| *Kdm6a*-F | 5’-ATCCCAGCTCAGCAGAAGTT-3’ |
| *Kdm6a*-R | 5’-GGAGGAAAGAAAGCATCACG-3’ |
| *Kdm6b*-F | 5’-CTCTGGAACTTTCATGCCGG-3’ |
| *Kdm6b*-R | 5’-CTTAGCCCCATAGTTCCGTTTG-3’ |
| *Bcl2*-F | 5’-TGGGATGCCTTTGTGGAACTAT-3’ |
| *Bcl2*-R | 5’-AGAGACAGCCAGGAGAAATCAAAC-3’ |
| *CD215*-F | 5’-AAGGAATGAAAAGGGAGATCG-3’ |
| *CD215*-R | 5’-AATTCTTGACCCGGATGTCA-3’ |
| *CD122*-F | 5’-CAGGACTGCAGGGAACATCT-3’ |
| *CD122*-R | 5’-GACAGGCTCCAGGGAAGAG-3’ |
| *CD132*-F | 5’-ACAGAGATCGAAGCTGGACG-3’ |
| *CD132*-R | 5’-GAACCCGAAATGTGTACCGT-3’ |
| *Tgfbr1*-F | 5’- CTCCTCATCGTGTTGGTGG-3’ |
| *Tgfbr1*-R | 5’- GCAAAGACCATCTGTCTCACA-3’ |
| *Tgfbr2*-F | 5’-CCGCTGCATATCGTCCTGTG-3’ |
| *Tgfbr2*-R | 5’-TGGATGCATCTTTCTGGGCTT-3’ |
| *Gzmb*-F | 5’-CCTGCTACTGCTGACCTTGT-3’ |
| *Gzmb*-R | 5’-CGAATAAGGAAGCCCCCACA-3’ |
| *Klra5*-F | 5’-TCACTGTGAGATCGCTTGGA-3’ |
| *Klra5*-R | 5’-TGTCACTTTGCATGTTGCTGC-3’ |
| *Klra6*-F | 5’-ATCACTCCGGTAGAGACACAGAGA-3’ |
| *Klra6*-R | 5’-TGCCAACACTGCAACTGTTAT-3’ |
| *Klra7*-F | 5’-ATAGAGTGTAGGCCAGGCAATG-3’ |
| *Klra7*-R | 5’-CTCTGCCTTGGAGATGGGTCT-3’ |
| *Klrc1*-F | 5’-CTCCTCCTTTGGCCTCATCA-3’ |
| *Klrc1*-R | 5’-GCTTCGGTATCTTTGCAGGG-3’ |
| *Klrd1*-F | 5’-TCTAGGATCACTCGGTGGAGA-3’ |
| *Klrd1*-R | 5’-CACTTGTCCAGGCAAACACAG-3’ |
| *Klre1*-F | 5’-TCCCTCTTTGCTTCAGCATTTT-3’ |
| *Klre1*-R | 5’-CCTCTGCTCAATGGATGACA-3’ |
| *Klri1*-F | 5’-ACCTACAGTCAAGCAGAGCC-3’ |
| *Klri1*-R | 5’-TCCAAGCATGCCAGTTACCA-3’ |
| *S1pr1*-F | 5’-CCTCTCGGACCTATTAGCAGGC-3’ |
| *S1pr1*-R | 5’-CGCAGAAACCACTGGGCAG-3’ |
| *CD160*-F | 5’-GGGGCTAATACTCTTCTGGTGC-3’ |
| *CD160*-R | 5’-CTTTTCAGTGATGCCATCTGTCT-3’ |
| *Fasl-*F | 5’-ACAACACAAATCTGTGGCTACC-3’ |
| *Fasl-*R | 5’-GGTGTACTGGGGTTGGCTAT-3’ |
| *Prf1-*F | 5’-TTCGGGAACCAAGCTACACC-3’ |
| *Prf1-*R | 5’-AGTAATGTGTGCAGGGGCTC-3’ |
| *GAPDH*-F | 5’-TTGACCTCAACTACATGGTCTA-3’ |
| *GAPDH*-R | 5’-ACCAGTAGACTCCACGACATAC-3’ |
| *CD8a-F* | 5’-CCGTTGACCCGCTTTCTGT-3’ |
| *CD8a-R* | 5’-CGGCGTCCATTTTCTTTGGAA-3’ |
| *CD103-F* | 5’-GCTGCATCTGCTCCAGCTAT-3’ |
| *CD103-R* | 5’-GCCCAGTCCACATCCATATT-3’ |
| *Tbx21-F* | 5’-TCAACCAGCACCAGACAGAG-3’ |
| *Tbx21-R* | 5’-ATCCTGTAATGGCTTGTGGG-3’ |
| *Runx3-F* | 5’-ACTTCCTCTGCTCCGTGCT-3’ |
| *Runx3-R* | 5’-GGTCACCACCGTTCCATC-3’ |
| *Thpok-F* | 5’-TTCTTCCTACACCCTGTGCC-3’ |
| *Thpok-R* | 5’-ATGGGATTCCAATCAGGTCA-3’ |
| *Il10-F* | 5’-ATCGATTTCTCCCCTGTGAA-3’ |
| *Il10-R* | 5’-TGTCAAATTCATTCATGGCCT-3’ |
