# Supplementary material for: The histone demethylase Kdm6b regulates the maturation and cytotoxicity of TCRαβ+CD8αα+ intestinal intraepithelial lymphocytes
Source: Cell Death Differ. 2022 Jan 9;29(7):1349–63. doi: 10.1038/s41418-021-00921-w (PMC9287323; doi:10.1038/s41418-021-00921-w)
Supplement: Supplementary file 12 — Supplementary Table 2 [file 41418_2021_921_MOESM12_ESM.docx]

| Supplementary Table 2. Primers used for ChIP-qPCR | |
| --- | --- |
| Gene name | Sequence |
| *Gzmb* promoter-F | 5’-CTGTAAGTGGTGGTTCTC-3’ |
| *Gzmb* promoter-R | 5’-TCTCTGCTTTTATGATGCT-3’ |
| *Bcl2* ERE enhancer-F | 5’-CACCTGTGGTCCATCTGAC-3’ |
| *Bcl2* ERE enhancer-R | 5’-CTGAAGAGTTCCTCCACCAC-3’ |
| *Fasl* promoter-F | 5’-GATGTTCAGGGAAGGGAC-3’ |
| *Fasl* promoter-R | 5’-GGGGAAAAGACCAAGGAG-3’ |
| *Ccr9* promoter-F | 5’-TGTGATTCCAAGCCCAAAC-3’ |
| *Ccr9* promoter-R | 5’-AAGTTCCCGACCGCAAG-3’ |
| *Klrd1* promoter-F | 5’-CTGCTTTTAGGGACGACTT-3’ |
| *Klrd1* promoter-R | 5’-AATCATCTGTTGGTGGGAC-3’ |
| *Klre1* promoter-F | 5’-ATGGGTGCTAAACGGAAAT-3’ |
| *Klre1* promoter-R | 5’-GAACTGGGAACAGGGGAGG-3’ |
| *Tbx21 promoter-F* | 5’-GAATTCGCGCTGTATTAGCC-3’ |
| *Tbx21 promoter-R* | 5’-GCCTTTGCTGTGGCTTTATG-3’ |
| *CD8a promoter-F* | 5’-CACTCAACCTCCAGACAA-3’ |
| *CD8a promoter-R* | 5’-GACTCACTTCATGGCAAA-3’ |
| *E8I enhancer-F* | 5’-TGGTTGCCTCTGCTACTT-3’ |
| *E8I enhancer-R* | 5’-CACATTCGCATTGGTTT-3’ |
| *Bcl2 promoter-F* | 5’-GCTCAGAGGAGGGCTTTCT-3’ |
| *Bcl2 promoter-R* | 5’-CCGGCCTCTTACTTCATTCT-3’ |
