# Supplementary material for: The histone demethylase Kdm6b regulates the maturation and cytotoxicity of TCRαβ+CD8αα+ intestinal intraepithelial lymphocytes
Source: Cell Death Differ. 2022 Jan 9;29(7):1349–63. doi: 10.1038/s41418-021-00921-w (PMC9287323; doi:10.1038/s41418-021-00921-w)
Supplement: Supplementary file 13 — Supplementary Table 3 [file 41418_2021_921_MOESM13_ESM.docx]

Supplementary Table 3. Primers used for intestinal microbiota analysis

| Gene name | Sequence |
| --- | --- |
| Universal bacterial-F | 5’-ACTCCTACGGGAGGCAGCAGT-3’ |
| Universal bacterial-R | 5’-ATTACCGCGGCTGCTGGC-3’ |
| Bacteroidetes-F | 5’-CATGTGGTTTAATTCGATGAT-3’ |
| Bacteroidetes-R | 5’-AGCTGACGACAACCATGCAG-3’ |
| Actinobacteria-F | 5’-CGCGGCCTATCAGCTTGTTG-3’ |
| Actinobacteria-R | 5’-ATTACCGCGGCTGCTGG-3’ |
| Firmicutes-F | 5’-GCTGCTAATACCGCATGATATGTC-3’ |
| Firmicutes-R | 5’-CAGACGCGAGTCCATCTCAGA-3’ |
| Proteobacteria-F | 5’-CATGACGTTACCCGCAGAAGAAG-3’ |
| Proteobacteria-R | 5’- CTCTACGAGACTCAAGCTTGC-3’ |
| Alphaproteobacteria-F | 5’- ACTCCTACGGGAGGCAGCAG-3’ |
| Alphaproteobacteria-R | 5’-TCTACGRATTTCACCYCTAC-3’ |
| Betaproteobacteria-F | 5’-ACTCCTACGGGAGGCAGCAG-3’ |
| Betaproteobacteria-R | 5’-TCACTGCTACACGYG-3’ |
| Epslionproteobacteria-F | 5’- TGGCGSACGGGTGAGTAATRTATAG-3’ |
| Epslionproteobacteria-R | 5’-GGAGTTTACRCWCCGAAAWGYGTC-3’ |
| Gammaproteobacteria-F | 5’- CMATGCCGCGTGTGTGAA-3’ |
| Gammaproteobacteria-R | 5’-ACTCCCCAGGCGGTCDACTTA-3’ |
| Enterobacteriaceae-F | 5’-GTGCCAGCMGCCGCGGTAA-3’ |
| Enterobacteriaceae -R | 5’-GCCTCAAGGGCACAACCTCCAAG -3’ |
| Bifidobacterium-F | 5’-CGGGTGAGTAATGCGTGACC-3’ |
| Bifidobacterium-R | 5’-TGATAGGACGCGACCCCA-3’ |
| Bacteroides-F | 5’-GGTTCTGAGAGGAGGTCCC-3’ |
| Bacteroides-R | 5’-GCTGCCTCCCGTAGGAGT-3’ |
| Bacteroides vulgatus-F | 5’-GCATCATGAGTCCGCATGTTC-3’ |
| Bacteroides vulgatus-R | 5’-TCCATACCCGACTTTATTCCTT-3’ |
| Bacteroides thetaiotaomicron-F | 5’-GGCAGCATTTCAGTTTGCTTG-3’ |
| Bacteroides thetaiotaomicron-R | 5’-GGTACATACAAAATTCCACACGT -3’ |
| Bacteroides uniformis-F | 5’- TCCGTTTTCCACTTATAAGA -3’ |
| Bacteroides uniformis-R | 5’- GGGTTBCCCCATTCGG -3’ |
| Prevotella-F | 5’-CACRGTAAACGATGGATGCC-3’ |
| Prevotella-R | 5’-GGTCGGGTTGCAGACC -3’ |
| Clostridial cluster IV-F | 5’-GCACAAGCAGTGGAGT-3’ |
| Clostridial cluster IV-R | 5’-CTTCCTCCGTTTTGTCAA-3’ |
| Clostridial cluster XIVa-F | 5’-AAATGACGGTACCTGACTAA-3’ |
| Clostridial cluster XIVa-R | 5’-CTTTGAGTTTCATTCTTGCGAA-3’ |
| Enterococcus-F | 5’-CCCTTATTGTTAGTTGCCATCATT-3’ |
| Enterococcus-R | 5’-ACTCGTTGTACTTCCCATTGT-3’ |
| Lactobacillus-F | 5’-AGCAGTAGGGAATCTTCCA-3’ |
| Lactobacillus-R | 5’- CACCGCTACACATGGAG-3’ |
| E.coli-F | 5’-CATGCCGCGTGTATGAAGAA-3’ |
| E.coli-R | 5’-CGGGTAACGTCAATGAGCAAA -3’ |
